# Processing in working memory boosts long-term memory representations and their retrieval

**DOI:** 10.1038/s44271-025-00309-3

**Published:** 2025-08-26

**Authors:** Melinda Sabo, Daniel Schneider

**Affiliations:** 1 Leibniz Research Centre for Working Environment and Human Factors, Dortmund, Germany; 2 Department of Psychology, Yale University, New Haven, CT USA

**Keywords:** Human behaviour, Attention, Long-term memory, Working memory

## Abstract

Prior research has explored how working memory influences the formation of new long-term memories, but its role in modifying existing representations remains unclear. This study examines whether attentional prioritization and testing in working memory enhance long-term memory retrieval and investigates the underlying neural mechanisms. Eighty-six participants completed a three-phase memory task combining a long-term memory—with a working memory retro-cue paradigm. First, participants learned object-location associations. Next, during a working memory task, some objects have undergone attentional prioritization and testing, others have only been tested in working memory. Finally, participants retrieved the object locations from long-term memory. Three key findings emerged: (1) both attentional prioritization and testing in working memory improved long-term memory retrieval; (2) serving as a probe in working memory further contributed to long-term memory enhancement, with benefits observed at behavioral and neural levels; and (3) cross-phase decoding revealed a comparable representational format for location information across task phases, possibly explained by the neural reinstatement of location information across phases. These results suggest that working memory dynamically shapes long-term memory representations, playing a more active and integrated role in long-term memory formation than previously thought.

## Abstract (web summary)

Attentional prioritization and testing in working memory not only effect immediate memory recall but can also enhance long-term memory and its underlying neural representation.

## Introduction

Understanding the interplay between short-term and long-term memory is crucial for studying human memory and learning. As early as the 1960s, Atkinson and Shiffrin proposed a continuous interaction between these memory systems^1^, a concept still central today. For instance, Cowan’s influential theory suggests a continuous information exchange between the two memory systems, whereby information in working memory activates long-term memory representations^2,3^, while retrieved long-term memory content is temporarily maintained in working memory^4^. Likewise, Oberauer and colleagues discuss the existence of a flexible gate that regulates the entry of long-term memory content into working memory, allowing it only when beneficial^5–8^.

Research has extensively examined how working memory processes influence long-term memory formation and recall. A key finding is the McCabe effect, in which long-term recall is enhanced for word lists interrupted by an arithmetic task during working memory maintenance compared to uninterrupted ones (complex vs simple span tasks)^9^. Previous research offers several explanations for this improvement, including retrieval during the arithmetic task^9–11^, extended time in working memory^12,13^, or elaboration during the maintenance period^14^.

Building on these findings, research has further explored how attention shapes the information transfer from working memory to long-term memory. Researchers have examined how attentional refreshing, value-based prioritization, and cue-based prioritization in working memory affect long-term memory formation. Attentional refreshing, which involves reactivating a recent stimulus^15^, has shown mixed results in terms of long-term recall: while some studies have reported benefits^16,17^, others have failed to replicate these effects^18,19^. A similar pattern has been observed with reward-based prioritization, where the results are inconsistent^20,21^. In contrast, cue-based attentional prioritization has been consistently shown to enhance long-term recall, demonstrating the most robust effect^20,22–25^.

These findings highlight how attentional processes in working memory impact the formation of long-term memories. However, an important question remains: Can working memory processes also enhance newly formed long-term memory representations? If so, what are the underlying neural mechanisms? The current study aims to address these questions. Specifically, it explores the relationship between working memory processes and long-term memory retrieval in a series of two experiments involving a total of 86 participants. Our first aim was to understand whether attentional prioritization and testing in working memory can boost the retrieval of newly formed long-term memory representations. Second, we sought to investigate the neural mechanisms underlying such enhancement effects. Theoretical accounts suggest that these benefits could arise through different pathways: either by forming additional memory traces each time the information is re-encountered in working memory^26^ or by gradually strengthening an existing trace.

**Fig. 1:**
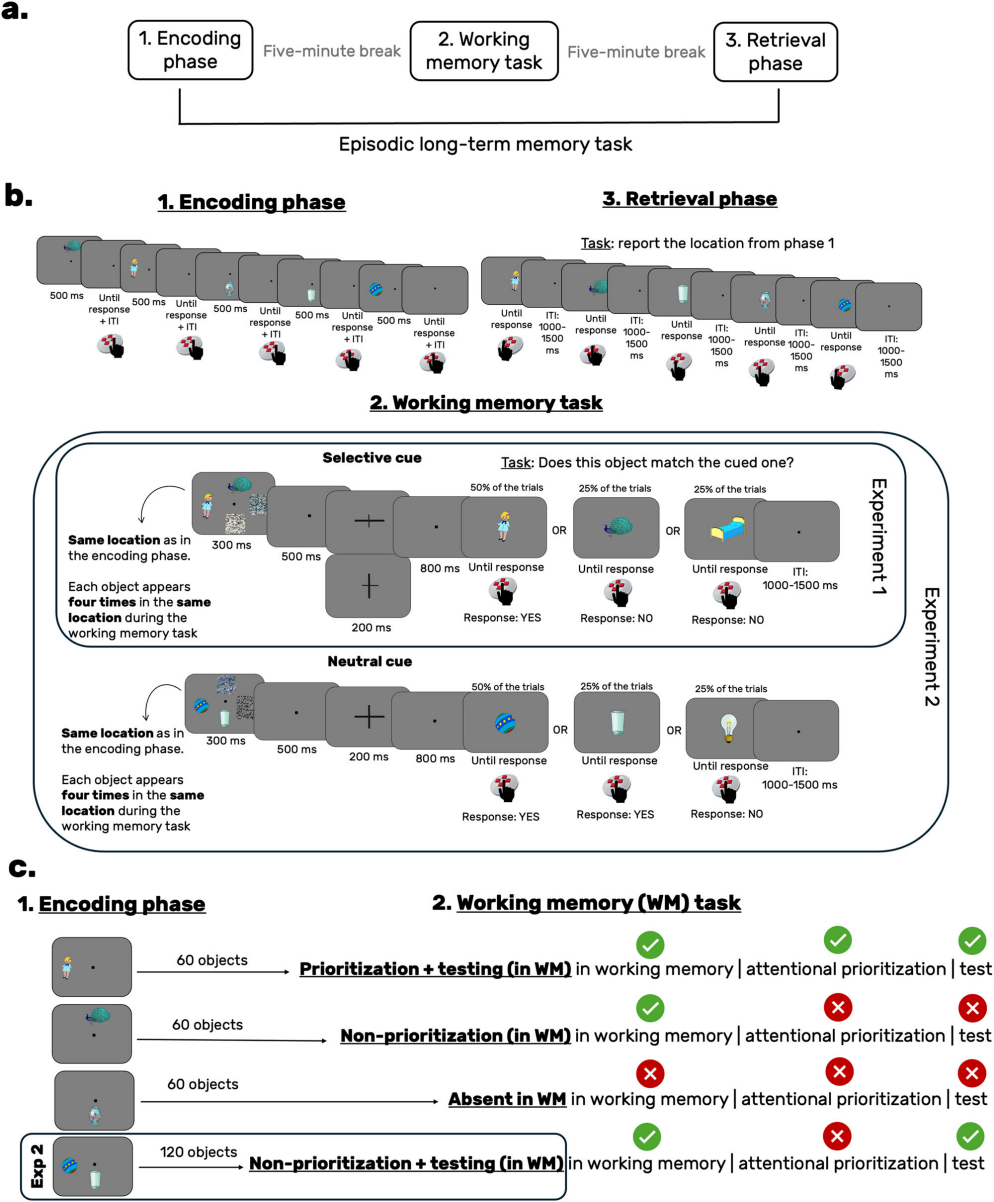
Overview of the experimental design. **a** The three main phases of the experiment. **b** A detailed overview of these phases. In Experiment 1, the working memory phase includes only selective (informative) retro-cues, whereas Experiment 2 incorporates both selective cues and neutral (non-informative) cues. **c** An overview of the working memory manipulation and the corresponding conditions. Panel c emphasizes how the same objects from the encoding phase reappear during the working memory task, undergoing different types of processing depending on their assigned condition. Importantly, in the *absent in working memory* condition, the object is not presented during the working memory task, appearing only during encoding and the final retrieval phase. Notably, the *non-prioritization + testing* condition is exclusive to Experiment 2, whereas the other three conditions are common to both experiments.

In Experiment 1, participants completed a combined episodic long-term and working memory task with three phases (see Fig. 1a, b). In the first phase, they learned object-location associations with the instruction to remember them for later recall. In the second phase, a working memory retro-cue paradigm was introduced. Participants were shown objects from the first phase, presented in their learned locations, along with two task-irrelevant scrambled objects. Participants’ task was to memorize the objects for a subsequent test. After a short maintenance period, a retro-cue indicated which object would be tested. During the test, participants had to decide if the cued object matched a centrally presented object. Each object appeared four times in the same location during the working memory task. In the final retrieval phase, participants were shown all objects from the first phase and were asked to report the initially learnt locations using a response device with four spatially aligned buttons corresponding to the four possible locations (see Fig. 1b). All analyses were performed on data collected during the long-term memory retrieval phase. Importantly, retrieval phase trials were categorized based on the processing that took place during the working memory task (for an overview of the conditions, see Fig. 1c). Experiment 1 includes three experimental conditions: (i) the *prioritization* + *testing* condition involves attentional prioritization and testing in working memory; (ii) the *non-prioritization* condition includes a brief presentation and maintenance of the object in working memory; and (iii) the *absent in working memory* (control) condition entails the absence of the object from the working memory task. According to our hypothesis, if attentional prioritization in working memory boosts long-term memory retrieval, we should observe improved behavioral performance and a stronger parietal old-new effect^27–31^ in the *prioritization* + *testing* condition compared to the *non-prioritization* and *absent in working memory* conditions. This would suggest that cue-based attentional prioritization in working memory can still enhance the retrieval of newly formed long-term memory representations. In Experiment 2, we adopted the same design, but introduced a new type of cue in the working memory task: uninformative/neutral cues. These cues did not provide information about the to-be-tested item, requiring participants to maintain both objects for the working memory test. We refer to this new condition as the *non-prioritization + testing* condition as  objects do not undergo attentional prioritization, but they are tested in working memory. By comparing the long-term memory retrieval performance between the non-prioritization + testing and the prioritization + testing conditions, we can determine whether the benefits observed in Experiment 1 are due solely to attentional prioritization or a combination of attentional prioritization and testing. Overall, understanding these processes both at the behavioral and neural level is essential, as it challenges the view of working memory as merely a temporary buffer and instead positions it as a dynamic interface, where long-term memory representations can be actively modified.

## Methods

### Participants—Experiments 1 and 2

In Experiment 1, data were collected from 49 participants, with six excluded: four due to EEG recording failures resulting in data loss, and two due to misunderstanding the task. One person consistently pressed the right button during the final retrieval phase, while the second one  did not understand the task instructions (as documented by the research team during data collection in accordance with the study protocol). The final sample included 43 participants (25 women, 18 men; gender information provided by participants), aged 18–31 years (*M*_age_ = 23.65, SD = 2.85). In Experiment 2, data from 45 participants were collected, with two excluded—one person for performing at chance level (25%) and the other one for reaction times six standard deviations (SDs) from the group mean. The final sample comprised 43 participants (25 women, 18 men; gender information provided by participants), aged 20–34 years (*M*_age_ = 24.97, SD = 3.94). Participants in both experiments were recruited from the Rhein-Ruhr region of Germany via online platforms such as Moodle and Facebook. Thus, a convenience sampling approach was used. Most participants were students or recent university graduates. No data on race or ethnicity were collected in either experiment. Data collection for both experiments spanned one year, from December 2022 to December 2023, with a four-month break between Experiments 1 and 2. None of the experiments were preregistered.

The sample size of 43 participants was determined based on a previous long-term memory study conducted by our group^32^,  providing a balance between statistical power and cost efficiency. All participants were right-handed, had normal or corrected vision, and no history of neurological or psychiatric disorders. Compensation was 12 €/h or study credits. Written consent was obtained, and the study was approved by the ethics committee of the Leibniz Research Centre for Working Environment and Human Factors (Dortmund, Germany).

### Experimental procedure—Experiments 1 and 2

Upon arrival, participants received an overview of the study and provided written consent. They then completed a demographic survey and the Edinburgh Handedness Inventory^33^. After EEG cap preparation, participants were guided into a dimly lit EEG laboratory. The experiment was presented on a 22-inch CRT monitor (100 Hz, 1024 × 768 pixels) at a viewing distance of ~145 cm, and it was programmed using the ViSaGe MKII Stimulus Generator (Cambridge Research Systems, UK) and Lazarus IDE (Free Pascal). The procedure began with a brief training session simulating the main task, followed by the main experiment, which included three phases: encoding, a working memory task, and retrieval (Fig. 1a), with 5-min breaks between phases. At the end of the study, participants completed a follow-up questionnaire about strategies and difficulties encountered (see Supplementary Materials, Note 6, for responses). All questionnaires were administered in a paper-and-pencil format.

### Experimental design—Experiment 1

During the encoding phase, participants viewed everyday objects at one of four screen locations: top, bottom, left, or right, while keeping their gaze on a central dot. They were instructed to memorize the object’s location by visualizing it after it disappeared, without using verbal repetition or semantic strategies. After each trial, participants confirmed memorization by clicking the right button to start the next trial. Each object appeared twice in the same location, with all 180 objects shown once before any repetition. The encoding phase contained 360 trials in total (see a detailed overview of the parameters, such as object size, fixation size, distance between object and fixation etc., in the Supplementary Materials, Note 1).

In the second phase, a working memory retro-cue paradigm was adopted (Fig. 1b). Participants were presented with a memory display that contained two objects from the encoding phase and two scrambled, task-irrelevant objects around a fixation dot. The intact objects appeared in their original, encoding phase locations, and participants memorized them for an immediate report. After a 500 ms maintenance period, a retro-cue indicated whether the object along the vertical or horizontal axis would be tested. Following an 800 ms interstimulus interval, a central probe appeared, and participants indicated ifthe probe matched the cued object by pressing the top/bottom button for yes and the other button for no. The response mapping was counterbalanced across participants. The probe matched the cued object 50% of the time, it was a non-cued object 25% of the time, and a new object 25% of the time, requiring a ‘no’ response on 50% of trials. Each object, bound to its location and condition, appeared four times during this phase, resulting in 240 trials in total.

In the final retrieval phase, participants were shown all objects from the encoding phase and reported each object’s location using a response device with four buttons corresponding to top, bottom, left, and right. They were instructed to keep their index finger in a neutral position (the center of the response button) between trials. Each object’s location was reported twice, resulting in 360 trials (180 objects, each presented twice).

Stimuli were selected from a stimulus set comprising everyday objects^34^. Although the original pool included 260 objects, our selection was refined based on a prior image rating survey^32^, in which we selected a subset of 240 objects exhibiting optimal luminance, contrast, vividness, and recognizability. Thus, for each participant, a random subset of 180 images was selected.

### Experimental conditions—Experiment 1

The special feature of the current experiment is that object-location associations initially encoded in phase 1, reappear in the second phase (i.e., in the working memory task). One-third of the objects consistently appear as a cued object, another one-third appears as a non-cued/non-prioritized item, while the remaining one-third does not reappear in the working memory task (see Fig. 1c). Objects were randomly assigned to these conditions. In each trial, a prioritized and a non-prioritized object were randomly paired, ensuring one appeared on the horizontal axis and the other on the vertical axis.

Experiment 1 includes three experimental conditions based on how objects were processed in working memory (or if they were excluded from such processing): (i) the *prioritization + testing* condition, involves attentional prioritization and testing of previously formed long-term memory representation in working memory; (ii) the *non-prioritization* condition, includes a brief presentation and maintenance of the object followed by the item being placed outside the focus of attention, as it is no longer relevant for the subsequent working memory test ; and (iii) *absent in working memory* (control) condition, in which the object is initially encoded into long-term memory and it is tested in the retrieval phase, but it does not appear in the working memory task (see Fig. 1c).

### Experimental design—Experiment 2

As outlined in the Introduction, Experiment 2 introduces a new type of cue in the working memory task: uninformative/neutral cues. Similar to Experiment 1, participants encoded the stimulus display and were probed in a match-no-match task at the end of the trial. However, unlike informative cues, neutral cues did not provide information about the to-be-tested item, requiring participants to maintain both objects for the comparison in the working memory test (see Fig. 1b). Similar to Experiment 1, participants did not report any locations during the working memory task. Since Experiment 2 contained a new condition, which required significantly more objects (i.e., 300 in total), stimuli from the pool introduced by Brady and colleagues^35^ were used. In total, 300 objects were manually selected. This resulted in 600 trials in the encoding and retrieval phase, each and 480 trials in the working memory task. All other aspects were kept consistent with Experiment 1.

### Experimental conditions—Experiment 2

In Experiment 2, objects were equally divided into the four conditions. Experiment 2 assumes that objects in the *prioritization + testing* condition undergo both attentional prioritization and testing in working memory. In contrast, objects in the *non-prioritization + testing* condition undergo non-selective maintenance and testing at the end of the trial. The other two conditions (*non-prioritization in working memory* and *absence in working memory*) are identical to Experiment 1.

### EEG recording

The EEG data were recorded using a passive 64 channel Ag/AgCl system (Easycap GmbH, Herrsching, Germany). The electrodes were distributed according to the extended 10/20 system^36^. During the EEG recording, a 250 Hz low-pass filter was applied, and the signal was amplified using a NeuroOne Tesla AC-amplifier (Bittium Biosignals Ltd, Kuopio, Finland). The obtained data has a sampling rate of 1000 Hz. FCz served as the reference electrode, while AFz served as the ground electrode. During the recording, the impedances were kept below 20 kΩ.

### Data analyses

MATLAB® (R2021b) was utilized for conducting all behavioral, EEG, and statistical analyses. Initially, the dataset of each participant was segmented into three phases, corresponding to the phases of the task.. Since we were interested in the consequences of differential processing in working memory, behavioral and EEG data from the retrieval phase were analyzed. Importantly, items were tested twice during the retrieval phase to increase the trial numbers and improve the signal-to-noise ratio. As accuracy did not differ between repetitions (see Supplementary Materials, Note 7), data from both tests were included consistently in all analyses.

### Behavioral analyses—Experiments 1 and 2

To answer our main research question, accuracy and response times recorded during the retrieval phase were assessed. These two parameters were contrasted between the three experimental conditions of Experiment 1 (i.e., *prioritization+testing, non-prioritization, and absent in working memory* conditions) and four experimental conditions of Experiment 2 (i.e., *prioritization + testing, non-prioritization + testing*, *non-prioritization*, and *absent in working memory* conditions). Additionally, for Experiment 2, we also contrasted the accuracy and response times relative to the number of instances, in which the respective object was presented as a central probe during the working memory task. Three categories were distinguished: tested zero times, once, and twice. Statistical comparisons were conducted using a repeated measures analysis of variance (rm-ANOVA) with the within-subject factor condition. Since Experiment 2 included more objects and thus participants were required to learn more associations, we conducted a control analysis to ensure that performance in Experiments 1 and 2 was comparable (see Supplementary Materials, Note 3 and Table S3).

### Preprocessing—Experiments 1 and 2

**Fig. 2:**
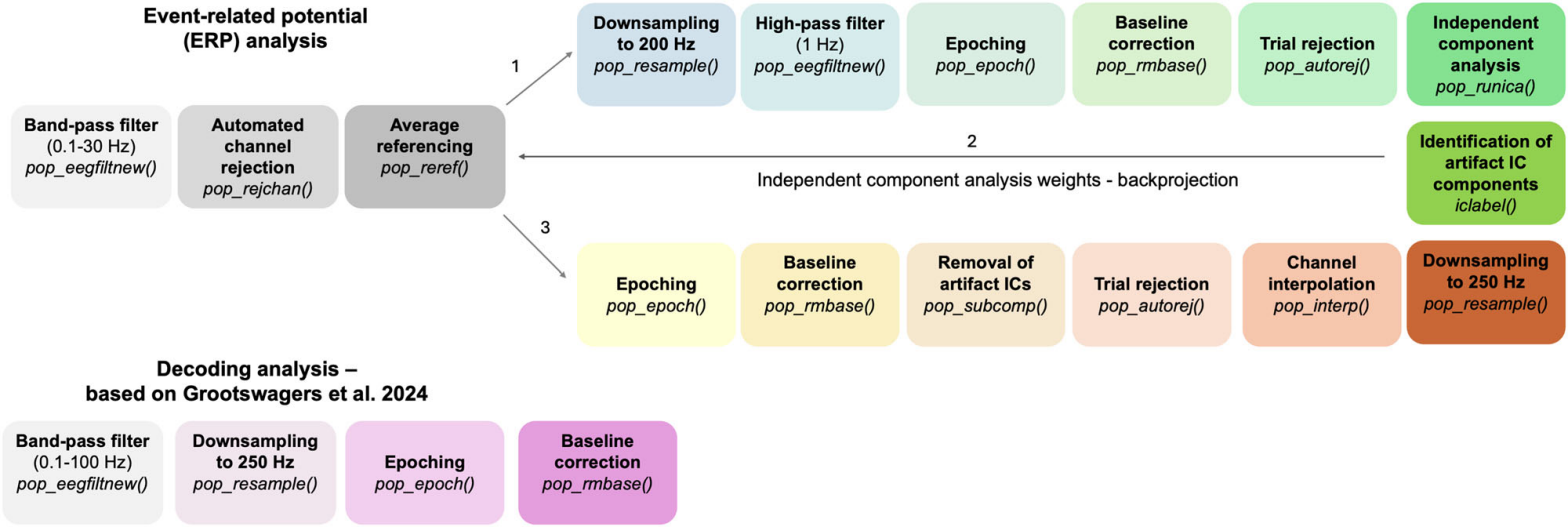
Overview of the preprocessing steps adopted for the ERP and decoding analyses.

The EEG data of Experiments 1 and 2 underwent preprocessing using the EEGLAB toolbox (version 14.1.2b;^37^) implemented in MATLAB®. The first step of the preprocessing pipeline was the application of a 0.1 Hz Hamming windowed sinc FIR high-pass filter (filter order: 33001, transition bandwidth: 0.1 Hz, cutoff frequency at −6 dB: 0.05 Hz) and a 30 Hz low-pass filter (filter order: 441, transition bandwidth: 7.5 Hz, cutoff frequency at −6 dB: 33.75 Hz) using the pop_eegfiltnew function. Following this, channels exhibiting substantial noise were identified and discarded through the automated channel rejection procedure in EEGLAB (for an overview, see Table 1) (pop_rejchan function). As a next step, the data were re-referenced to the average. Notably, channels capturing anterior eye movements (i.e., Fp1, Fp2, AF3, AF4, AF7, and AF8) were excluded from rejection to optimize eye-movement-related component detection in the subsequent independent component analysis (ICA). Preparing for ICA, the data were downsampled to 200 Hz to accelerate computation. Importantly, at the end of the preprocessing pipeline, the data were downsampled to 250 Hz to retain a higher temporal resolution (see Fig. 2). Next, the data were subjected to a 1 Hz Hamming windowed sinc FIR high-pass filter (pop_eegfiltnew, filter order: 661, transition bandwidth: 1 Hz, cutoff frequency at −6 dB: 0.5 Hz). Subsequently, epochs spanning −1000 ms to 3000 ms relative to object onset were established, followed by baseline correction (−200 ms to 0 ms). Trials exhibiting extreme fluctuations were then rejected via EEGLAB’s automated trial rejection procedure (for an overview, see Table 1) (pop_autorej; ‘threshold’: 500 μV; ‘Startprob’: SD value for flagging trials for rejection: 5 SDs; ‘maxrej’: maximum % of rejected trials: 5%). This function performs artifact rejection using a statistical approach that flags improbable epochs based on their deviation from the overall data. The ‘threshold’ parameter defines a high-amplitude cutoff for flagging candidate trials for rejection. ‘Startprob’ sets the SD threshold for outliers, and ‘maxrej’ limits rejections per iteration. This allows for adaptive, data-driven artifact detection beyond fixed amplitude criteria. Next, ICA was conducted on the rank-reduced data (remaining number of channels minus one, determined via principal component analysis within the pop_runica function). Identification of artifact-containing independent components (ICs) was realized via the ICLabel plug-in (version 1.3)^38^, which categorizes ICs into brain, muscle, eye, heart, line noise, channel noise, and other noise. Following the criteria established by Wascher and colleagues^39^, ICs labeled with ≥30% probability as eye movement components or <30% probability as brain components were discarded. Subsequently, the ICA weights were backprojected to the original 1000 Hz data (see Fig. 2). Data were afterwards epoched (−1000 ms to 3000 ms) and underwent baseline correction (−200 ms to 0 ms). ICs marked for rejection were removed, and trials with significant fluctuations were discarded using the same automated procedure mentioned above (for an overview, see Table 1) (‘threshold’: 1000 μV; ‘Startprob’: SD value for flagging trials for rejection: 5 SDs; ‘maxrej’: maximum % of rejected trials: 5%). Finally, missing channels were interpolated using the spherical spline method in EEGLAB (pop_interp).

**Table 1:** Overview of the outcome of each preprocessing pipeline—Experiments 1 and 2

Experiment	Rejected number of channels	Number of remaining trials before ICA	Number of rejected IC components	Number of remaining trials after the ICA
Experiment 1	*M* = 1.62 Range: 0–5	*M* = 311.34 (86.49%) Range: 253–348	*M* = 31.97 Range: 14–58	*M* = 312.95 (86.93%) Range: 239–358
Experiment 2	*M* = 1.72 Range: 0–5	*M* = 491.46 (82.02%) Range: 363–559	*M* = 32.46 Range: 19–46	*M* = 479.81 (80%) Range: 380–557

Since Experiment 2 also included a decoding procedure, the preprocessing pipeline has been adapted. Due to the inherent robustness of multivariate methods against noise^40^, only minimal preprocessing steps were applied in this case (cf., ^41^). The EEG data underwent high-pass and low-pass filtering using a Hamming-windowed sinc finite impulse response (FIR) filter before being downsampled to 250 Hz. Epochs were then extracted for each phase, time-locked to stimulus presentation, as follows: (i) −500 ms to 1000 ms for the encoding phase; (ii) −500 ms to 3000 ms for the working memory task; (iii) −500 ms to 2500 ms for the final retrieval phase. Finally, baseline correction was performed using a 0–200 ms pre-stimulus interval.

### Event-related component analysis—Experiments 1 and 2

To test our main hypothesis, we assessed the left parietal old-new ERP component, previously argued to reflect long-term memory recollection^27–31^. First, the data were downsampled to 250 Hz. Next, we extracted the ERPs relative to the experimental conditions, and the data were averaged across the left parietal electrode cluster (Pz, P1, P3, and P5)^42^. For the statistical comparison, a cluster-based permutation procedure was adopted. For Experiment 1, this procedure was applied three times for the following contrasts: (i) *prioritization + testing* vs *non-prioritization*; (ii) *prioritization + testing* vs *absent in working memory*; and (iii) *non-prioritization* vs *absent in working memory*. Experiment 2 included three additional contrasts: (i) *prioritization + testing* vs *non-prioritization+testing*; (ii) *non-prioritization + testing* vs *non-prioritization*; (iii) *non-prioritization + testing* vs *absent in working memory*. For each analysis, the time window was restricted to 0–1200 ms after object onset. The typical left-parietal old-new effect is usually observed between 500 ms and 800 ms^31,42^. However, because we employed cluster-based permutation statistics, the time window was extended to account for additional effects. Additionally, the same analyses were conducted to compare the parietal old-new effect across different conditions, specifically contrasting the number of probing instances (zero times, once, or twice).

The cluster-based permutation procedure employed in this study consisted of several steps aimed at identifying significant differences between conditions while controlling for multiple comparisons. First, the power values of each contrast were compared at each of the 875 time points using paired-sample *t*-tests. This initial step yielded a vector of 875 *p*-values. Next, clusters of contiguous time points with significant *p*-values (*p* < 0.05) were identified. A cluster was defined as a group of adjacent time points where the *t*-tests yielded significant results. Only clusters containing more than one significant *p*-value were retained for further analysis.

To assess the statistical significance of these clusters, a distribution of maximum cluster sizes under the null hypothesis was generated. This was achieved by randomly shuffling the condition labels (e.g., *absent in working memory* and *non-prioritization + testing* conditions) across 10,000 iterations. For each permutation, paired-sample *t*-tests were conducted across all time points, producing a new vector of 875 *p*-values for each iteration. Afterwards, the largest cluster of contiguous significant *p*-values (*p* < 0.05) was identified and its size recorded. This process resulted in a distribution of maximum cluster sizes obtained under the null hypothesis. The 95th percentile of this distribution was then calculated to establish a threshold for statistical significance. Clusters from the original data that exceeded this threshold were deemed significant.

### Decoding analyses—Experiment 2

In addition to the ERP analyses, multivariate pattern analyses were also conducted using the CoSMoMVPA toolbox^43^ within MATLAB. Across all analyses, the objective was to train and test a classifier to distinguish between four stimulus locations (i.e., left, right, top, and bottom), with a chance level of 25% (i.e., 0.25). Linear discriminant analysis was used as the classifier.

The first stage focused on within-phase decoding, where training and testing were conducted using trials from the same phase (i.e, encoding, working memory, retrieval phase). Training and testing were conducted separately at each time point. A 10-fold cross-validation scheme was employed, whereby the classifier was trained on 90% of the data and tested on the remaining 10%, ensuring that each segment served as the test set once. To prevent class imbalance, the cosmo_balance_partitions function was applied, ensuring equal representation of all four locations in both training and testing datasets. Additionally, to increase the robustness of the model, the data were trained and tested on trial averages (i.e, super-trials). Data from all 64 channels served as input features for the classifier. For every time point, decoding performance was computed as the proportion of correctly classified trials.

As a second step, we conducted a cross-phase decoding procedure, following the same approach as the within-phase decoding analysis. The key difference was that the EEG data were trained on one phase and tested on another. For the encoding phase, we selected the time window 0–500 ms. In the working memory task, the analysis window was restricted to 300–800 ms, as this period reflects the active maintenance of location information in working memory. For the retrieval phase, we focused on the 500–1000 ms window, based on previous research suggesting that retrieved long-term memory representations are reactivated approximately 500 ms after cue presentation^44^. For the statistical analyses, we applied the same cluster-based permutation approach used in the ERP analyses. The only difference in this case was that, instead of permuting condition labels, we generated the null distribution by randomly exchanging observed decoding accuracy values with the chance-level value (0.50) within each trial and time point, which resulted in two vectors. This manipulation was repeated across 10,000 iterations. For each iteration, paired-sample *t*-tests were conducted at each time point. Importantly, since we hypothesized that the decoding accuracy is higher than chance level, we applied one-sided *t*-tests. Clusters of contiguous time points with significant *p*-values (*p* < 0.05) were then identified, and the size of the largest cluster was recorded. Repeating this process across all permutations yielded a null distribution of maximum cluster sizes, representing the range of cluster sizes expected by chance when decoding performance does not exceed baseline. The 95th percentile of this distribution was used to define the cluster-level threshold for statistical significance. Clusters in the original data that exceeded this threshold were considered statistically significant. To enhance the robustness of the results, data from both phases were used for both training and testing sets in a reciprocal fashion.

### Statistical analyses—Experiments 1 and 2

We conducted our statistical analyses both using a frequentist and a Bayesian framework. The former analyses were conducted using the functions of the Statistics and Machine Learning Toolbox implemented in MATLAB® (R2021b). In case of the rm-ANOVA, we tested the data for normality using the Lilliefors test and for sphericity using Mauchly’s test. Although rm-ANOVA is generally considered robust to minor violations of normality, we took a conservative approach; if even one condition deviated from normality, we conducted an additional non-parametric Friedman test to confirm that our results are robust. When the assumption of sphericity was violated, we applied the Greenhouse–Geisser correction. Partial eta squared (ηp²) was calculated as the measure of effect size for the rm-ANOVA, while Kendall’s W was calculated as a measure of effect size for the Friedman test. Whenever the rm-ANOVA or the Friedman test indicated significant main effects, we conducted follow-up pair-wise comparisons using paired sample-*t*-tests, as well as the non-parametric Wilcoxon Signed-Rank test. Unless otherwise noted, two-tailed *p*-values were computed. Effect sizes for the paired-sample *t*-test were calculated using Cohen’s *d*_av_, following Lakens’ recommendations^45^, while in the case of the Wilcoxon Signed-Rank test, we used *r*, calculated as the *z*-value divided by the square root of the sample size. For the post-hoc tests requiring multiple comparison adjustments, false discovery rate^46^ corrected *p*-values (denoted as *p*_adj_) were provided.

The Bayesian statistical analyses were conducted in JASP (v.0.18.3)^47^. Within the Bayesian framework, Bayes factors denote the amount of evidence in favor of the alternative or the null hypothesis. BF_10_ represents evidence in favor of an effect or condition difference, while BF_01_ (1/BF_10_) denotes evidence for the lack of an effect or condition difference. Bayes factors lower than 3 have been interpreted as weak / inconsistent evidence, values between 3 and 20 as positive or substantial evidence, Bayes factors ranging between 20 and 150 as strong evidence, and finally values higher than 150 as very strong evidence^48^. For both the rm-ANOVA and the Bayesian *t*-tests, a multivariate Cauchly prior was adopted, as implemented in JASP, with all default settings and parameters.

### Reporting summary

Further information on research design is available in the Nature Portfolio Reporting Summary linked to this article.

## Results

### Long-term memory accuracy is boosted by attentional prioritization in working memory—evidence from Experiment 1

**Fig. 3:**
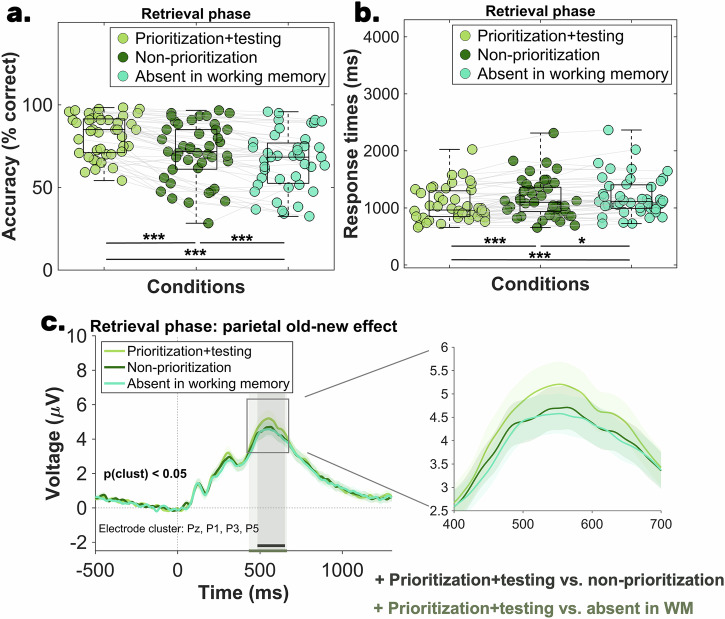
Results of Experiment 1—final retrieval phase. The scatterplots of (**a**, **b**) illustrate the mean accuracy and mean reaction time recorded during the three conditions of the final long-term memory retrieval phase (*N* = 43). The central mark in each boxplot represents the median, while the bottom and top edges denote the 25th and 75th percentiles, respectively. The whiskers do not extend to averages, which are treated as outliers. Gray lines connect data points from the same participant. Statistical significance is indicated with the following symbols: ^*^p < 0.05; ^**^p < 0.01, ^***^p < 0.001. **c** The parietal old-new effect recorded during the retrieval phase. The three shaded regions indicate the significant time window identified through the cluster-based permutation analysis: (i) 484-652 ms for the *prioritization + testing* vs *non-prioritization* contrast and (ii) 432-664 ms for the *prioritization + testing* vs *absent in working memory* condition contrast. The shaded area around the ERP of each condition denotes the standard error of the mean.

Table 2 shows the average retrieval phase accuracy, indicating that participants were most accurate in the *prioritization + testing* condition, followed by the *non-prioritization* condition, and least accurate in the *absent working memory* condition. Statistical comparisons using repeated measures ANOVA (rm-ANOVA) revealed that sphericity was violated (*χ*^2^(42)  =  7.58, *p*  =  0.02, ε  =  0.85), so Greenhouse–Geisser corrected results are reported. The accuracy analysis showed a main effect of condition: *F* (2, 84) = 51.85, *p*_*corr*_ <0 .001, η_p_^2^ = 0.55, *BF*_*10*_ = 1.98 × 10^12^, with the Bayes factor indicating very strong evidence in favor of the alternative hypothesis (see Fig. 3a). These results were also supported by the Friedman test, χ²(42) = 45.46, *p* < 0.001, Kendall’s W = 0.52. The Bayes factors of the post-hoc analyses provided very strong evidence for accuracy differences between all condition pairs, which was further supported by significant results from both paired *t*-tests and non-parametric tests: (i) *prioritization + testing* vs *non-prioritization* condition: *t*(42)  =   6.51, *p*_adj_  <  0.001, *d*_av_  =  0.70, 95% CI [7.29, 13.83], *BF*_*10*_ = 197994.04 and *W* = 905, *z* = 5.21, *p*_adj_  <  0.001, *r* = 0.79; (ii) *prioritization + testing* vs *absent in working memory* condition: *t*(42)  =   8.68, *p*_adj_  <  0.001, *d*_av_  =  1.05, 95% CI [12.10, 19.44], *BF*_*10*_ = 1.55 × 10^8^ and *W* = 805.50, *z* = 5.31, *p*_adj_  <  0.001, *r* = 0.81; (iii) *non-prioritization* vs *absent in working memory* condition: *t*(42)  =   4.19, *p*_adj_  <  0.001, *d*_av_  =  0.29, 95% CI [2.70, 7.72], *BF*_*10*_ = 178.07 and *W* = 760.50, *z* = 3.86, *p*_adj_  <  0.001, *r* = 0.58.

**Table 2:** Descriptive statistics—Experiment 1

Mean and SD					
Condition	*N*	Mean accuracy	SD accuracy	Mean response times	SD response times
*Prioritization + testing*	43	81.68%	12.39%	1060.80 ms	294.63 ms
*Non-prioritization*	43	71.12%	17.60%	1166.81 ms	342.96 ms
*Absent in working memory*	43	65.91%	17.58%	1206.22 ms	350.33 ms

These results indicate that cued attentional prioritization in working memory leads to an increase in long-term memory accuracy, surpassing performance for both items that were absent in the working memory task and those that were shortly presented and maintained in working memory (*non-prioritization* condition).

### Long-term memory retrieval speed is boosted by  attentional prioritization in working memory—evidence from Experiment 1

Descriptive analyses (Table 2) revealed that response times during long-term memory retrieval mirrored the accuracy pattern: participants responded fastest for objects in the *prioritization + testing* condition, followed by the *non-prioritization* condition, and slowest for the *absent in working memory* condition. A repeated measures ANOVA (rm-ANOVA) was conducted to contrast response times across conditions. Notably, all trials were included in the analysis regardless of accuracy. Since sphericity was violated (*χ*^*2*^(42)  =  14.03, *p*  <  0.001, ε  =  0.77), Greenhouse–Geisser corrected results are reported. Results of the rm-ANOVA revealed a main effect of condition: *F* (2, 84) = 27.32, *p*_*corr*_ < 0.001, η_p_^2^ = 0.39, *BF*_*10*_ = 1.17 × 10^7^, with the Bayes factor indicating very strong evidence in favor of the alternative hypothesis (see Fig. 3b). These results were also supported by the Friedman test, χ²(42) = 34.47, *p* < 0.001, Kendall’s W = 0.40. Post-hoc analyses (both paired *t*-tests and non-parametric tests) revealed significant results, as well as very strong evidence for the response times difference between: (i) *prioritization + testing* vs *non-prioritization* condition: *t*(42)  = -5.23, *p*_adj_  <  0.001, *d*_av_  =  0.33, 95% CI [−146.89, −65.12], *BF*_*10*_ = 3768.74 and *W* = 103, *z* = −4.46, *p*_adj_  <  0.001, *r* = −0.68; and (ii) *prioritization + testing* vs *absent in working memory* condition: *t*(42)  =  −5.89, *p*_adj_  <  0.001, *d*_av_  =  0.45, 95% CI [−195.21, −95.62], *BF*_*10*_ = 28713.80 and *W* = 83, *z* = −4.70, *p*_adj_  <  0.001, *r* = −0.71. Finally, the response times difference between the *non-prioritization* vs *absent in working memory* condition was supported by substantial evidence: *t*(42)  = −2.64, *p*_adj_  =  0.01, *d*_av_  =  0.11, 95% CI [−69.53, −9.29], *BF*_*10*_ = 3.50 and *W* = 250, *z* = −2.69, *p*_adj_  =  0.007, *r* = −0.41. Overall, similar to the accuracy results, the response times pattern also confirms that cued prioritization is associated with faster responses, which exceed the benefits observed when the item is either absent from working memory or briefly presented and maintained in working memory.

### Enhanced parietal old-new component for prioritized items in working memory—evidence from Experiment 1

In addition to the behavioral measures, we also explored whether differential processing in working memory impacts the amplitude of the parietal old-new effect. This event-related potential component is typically measured in tasks requiring old (previously encountered) vs new (never encountered) decisions during long-term memory retrieval and has been argued to reflect the conscious recollection of long-term memories^27,29–31^. Previous research has shown that the amplitude of this component varies depending on whether the information is deeply or superficially encoded^28^ or how precisely the information is remembered^42^. As indicated in Fig. 3c, data from the electrode cluster Pz, P1, P3, P5^42^ were averaged and contrasted between conditions via cluster-based permutation statistics. Our analysis revealed a significant cluster between 484-652 ms for the *prioritization + testing* vs *non-prioritization* contrast and a cluster between 432-664 ms for the *prioritization + testing* vs *absent in working memory* condition contrast. No statistically reliable effects were found when the *non-prioritization* vs *absence in working memory* conditions were compared. Importantly, although our primary analysis was based on a literature-driven electrode selection, we demonstrate that the findings are robust even when a broader electrode cluster is used (see Supplementary Materials, Note 5). Together, these results suggest that attentional prioritization in working memory enhances the neural representation of the retrieved content.

### Long-term memory accuracy is boosted by attentional prioritization above and beyond testing in working memory—evidence from Experiment 2

In Experiment 2, we aimed to disentangle the benefits of attentional prioritization and testing in working memory for subsequent long-term memory retrieval. To investigate this, a new working memory condition involving testing without prioritization, hereby referred to as the *non-prioritization + testing*, was introduced. Comparing this condition to the *prioritization + testing* condition allows us to isolate the added effect of attentional prioritization above and beyond testing in working memory.

**Fig. 4:**
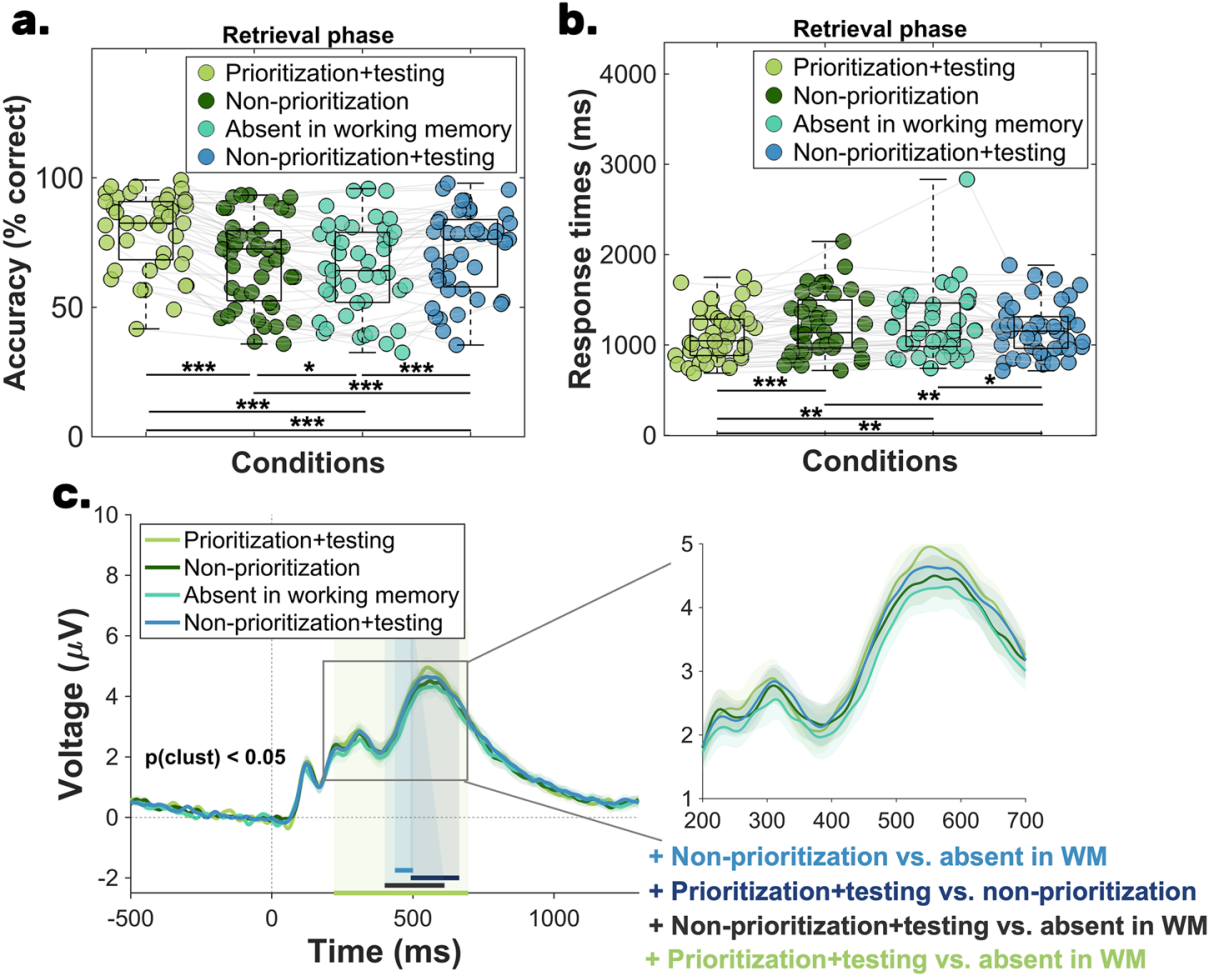
Behavioral and ERP results of Experiment 2—final retrieval phase (*N* = 43). The scatterplots of (**a**, **b**) illustrate the mean accuracy and mean reaction time recorded during the long-term memory retrieval phase. The central mark in each boxplot represents the median, while the bottom and top edges denote the 25th and 75th percentiles, respectively. The whiskers do not extend to averages, which are treated as outliers. Gray lines connect data points from the same participant. Statistical significance is indicated with the following symbols: ^*^p < 0.05; ^**^p < 0.01, ^***^p < 0.001. **c** The parietal old-new effect measured during the retrieval phase. The three shaded regions indicate the significant time windows identified through the cluster-based permutation analysis: (i) 492-664 ms for *prioritization + testing* vs *non-prioritization*, (ii) 220-696 ms for *prioritization + testing* vs *absent in working memory* condition, and (iii) 400-612 ms for *non-prioritization + testing* vs *absent in working memory* condition, (iv) 436-500 ms for *non-prioritization* vs *absent in working memory* condition. The shaded areas around the ERP of each condition denote the standard error of the mean.

At a descriptive level (see Table 3), participants were the most accurate in the *prioritization+testing* condition, followed by the *non-prioritization + testing* condition, then the *non-prioritization* condition, and finally the *absent in working memory* condition. To contrast participants’ accuracy, we conducted a rm-ANOVA. Because the sphericity assumption was violated (*χ*^*2*^(42)  =  21.42, *p*  < 0.001, ε  =  0.75), the Greenhouse–Geisser correction was applied. Results suggested a main effect of condition, which was also supported by the very strong evidence in favor of the alternative model as revealed by the Bayes factor: *F* (3, 126) = 63.76, *p*_*corr*_ < 0.001, η_p_^2^ = 0.60, *BF*_*10*_ = 6.40 × 10^21^ (Fig. 4a). A comparable result pattern was also revealed by the Friedman test, χ²(42) = 75.73, *p* < 0.001, Kendall’s W = 0.58. The Bayes factor of the post-hoc tests revealed very strong evidence for the accuracy difference between: (i) *prioritization + testing* vs *non-prioritization;* (ii) *prioritization + testing* vs *absent in working memory;* (iii) *prioritization + testing* vs *non-prioritization + testing*; (iv) *non-prioritization* vs *non-prioritization + testing; (v) absent in working memory vs non-prioritization + testing*. Finally, weak evidence was found for the contrast of *non-prioritization* vs *absent in the working memory* condition (Table 4).

**Table 3:** Descriptive statistics—Experiment 2

Mean and SD—Experiment 2					
Condition	*N*	Mean accuracy	SD accuracy	Mean response times	SD response times
Prioritization + testing	43	79.65%	14.53%	1108.37 ms	273.99 ms
Non-prioritization	43	67.75%	17.15%	1220.77 ms	342.00 ms
Absent in working memory	43	64.61%	17.78%	1229.77 ms	369.52 ms
Non-prioritization + testing	43	71.47%	16.46%	1164.54 ms	291.11 ms

**Table 4:** Accuracy results of Experiment 2—pairwise comparisons

Frequentist and Bayesian *t*-test results for accuracy—Experiment 2										
Condition contrast	d*f*	*t*	*p*_adj_	*d*_av_	95% CI	*BF*_*10*_	*W*	*z*	*p*_adj_	*r*
Prioritization + testing vs non-prioritization	42	11.26	<0.001	0.75	[9.76, 14.03]	2.55 × 10^11^	942	5.66	<0.001	0.86
Prioritization + testing vs absent in working memory	42	10.62	<0.001	0.93	[12.18, 17.89]	4.35 × 10^10^	896.50	5.56	<0.001	0.84
Prioritization + testing vs non-prioritization + testing	42	10.52	<0.001	0.52	[6.61, 9.74]	3.31 × 10^10^	900	5.60	<0.001	0.85
Non-prioritization vs absent in working memory	42	2.44	0.01	0.17	[0.54, 5.73]	2.33	595.50	1.80	0.07	0.27
Non-prioritization vs non-prioritization + testing	42	−4.18	<0.001	0.22	[−5.51, −1.92]	717.21	154	−3.72	<0.001	−0.56
Absent in working memory vs non-prioritization + testing	42	−5.16	<0.001	0.40	[−9.54, −4.18]	3090.97	107	−4.19	<0.001	−0.63

This suggests that both the *prioritization + testing* and the *non-prioritization + testing* conditions resulted in higher accuracy compared to the *absent in working memory* condition and the *non-prioritization* condition. However, accuracy was higher in the *prioritization + testing* condition compared to the *non-prioritization + testing* condition, which suggests a benefit of attentional *prioritization* over testing. It is important to note, however, that due to the structure

of the experimental design, we are specifically isolating the added benefit of attentional prioritization in conjunction with testing. Therefore, the observed advantage of prioritization over testing should not be interpreted as evidence that prioritization alone is superior to testing alone. Finally, these findings remain consistent even when the number of times an object appeared as the central probe object during the match-no-match task is controlled (see Supplementary Materials, Note 2 and Fig. S1).

### Long-term memory retrieval speed is boosted by attentional prioritization above and beyond testing in working memory—evidence from Experiment 2

As indicated in Table 3, at a descriptive level, participants were fastest in the *prioritization + testing* condition, followed by the *non-prioritization + testing* condition, the *non-prioritization* condition, and finally by the *absent in working memory* condition. We conducted an rm-ANOVA to investigate the effect of condition on long-term memory retrieval speed. 2.4. Sphericity was again violated (*χ*^*2*^(42)  =  30.13, *p*  <  0.001, ε  =  0.65), thus the Greenhouse–Geisser corrected results are reported: *F* (3, 126) = 9.94, *p*_*corr*_ < 0.001, η_p_^2^ = 0.19, *BF*_*10*_ = 2758.43. The Bayes factor revealed again very strong evidence for the alternative hypothesis, which predicts a main effect of condition (Fig. 4b). These results were also supported by the Friedman test, χ²(42) = 31.30, *p* < 0.001, Kendall’s W = 0.24.

The post-hoc tests revealed very strong evidence for the response times difference between the *prioritization+testing* vs the *non-prioritization* condition. Similarly, strong evidence was found for the difference between *prioritization + testing* vs *absent in working memory*. Furthermore, substantial evidence was found supporting the difference between the (i) *prioritization + testing* vs *non-prioritization + testing*; (ii) *non-prioritization* vs *non-prioritization + testing* comparisons. Finally, our analysis revealed substantial evidence for the lack of response times difference between the *non-prioritization* vs *absent in working memory* condition and weak evidence for the difference between *absent in working memory* condition vs *non-prioritization + testing* condition (Table 5).

**Table 5:** Response times results of Experiment 2—pairwise comparisons

Frequentist and Bayesian *t*-test results for response times—Experiment 2										
Condition contrast	d*f*	*t*	*p*_adj_	*d*_av_	95% CI	*BF*_*10*_	*W*	*z*	*p*_adj_	*r*
Prioritization + testing vs non-prioritization	42	−4.67	<0.001	0.36	[−160.97, −63.83]	701.88	133	−4.10	<0.001	−0.62
Prioritization + testing vs absent in working memory	42	−3.47	0.003	0.37	[−191.97, −50.83]	25.17	170	−3.65	<0.001	−0.55
Prioritization + testing vs non-prioritization + testing	42	−2.94	0.007	0.19	[−94.67, −17.67]	6.92	237	−2.84	<0.001	−0.43
Non-prioritization vs absent in working memory	42	−0.37	0.70	0.02	[−57.21, 39.22]	0.17 (BF_*01*_ = 5.66)	478	0.06	0.95	0.009
Non-prioritization vs non-prioritization + testing	42	3.10	0.006	0.17	[19.62, 92.83]	9.99	710	2.86	0.006	0.43
Absent in working memory vs non-prioritization + testing	42	2.37	0.02	0.19	[9.70, 120.75]	2.00	683	2.53	0.01	0.38

Overall, both attentional prioritization and testing in working memory led to faster long-term memory recall relative to the control condition (*absent in working memory*). Additionally, we also found a speed advantage of attentional prioritization over testing.

### Enhanced parietal old-new component for prioritized and tested items in working memory—evidence from Experiment 2

Similar to Experiment 1, we investigated the parietal old-new effect as an EEG correlate of long-term memory recollection. Data from the electrode cluster Pz, P1, P3, P5^42^ were averaged and contrasted across all six condition combinations using cluster-based permutation statistics. Importantly, although our primary analysis was based on a literature-driven electrode selection, we demonstrate that the findings are robust even when a broader electrode cluster is used (see Supplementary Materials, Note 5). The results revealed significant clusters between (i) 492-664 ms for *prioritization + testing* vs *non-prioritization*, (ii) 220-696 ms for *prioritization + testing* vs *absent in working memory condition*, and (iii) 400-612 ms for *non-prioritization + testing* vs *absent in working memory condition*, and (iv) 436-500 ms for *non-prioritization* vs *absent in working memory condition* (Fig. 4c). The other comparisons (*prioritization + testing* vs *non-prioritization + testing and non-prioritization* vs *non-prioritization + testing*) were not statistically significant. Overall, we were able to replicate the ERP results of Experiment 1: attentional prioritization led to a retrieval benefit relative to both control conditions (i.e., *non-prioritization* and *absent in working memory* conditions). In contrast, testing led to only an increase in the ERP effect relative to the *absence in the working memory* condition. Finally, at the neural level, the effects of attentional prioritization and testing on the subsequent long-term memory retrieval were equivalent.

### Long-term memory retrieval is boosted when the item serves as a probe multiple times in working memory—evidence from Experiment 2

As highlighted in the previous sections, testing in working memory enhanced long-term memory retrieval, even though location information became irrelevant early in the working memory trial. To better understand the source of this benefit, we conducted exploratory behavioral and EEG analyses focused on the number of probing instances during the working memory task. Specifically, we examined how often a particular object appeared as a probe (i.e., in the center of the screen during the final test of the working memory task) within the *non-prioritization + testing* condition. We hypothesized that encountering a familiar object as the central probe during the working memory task might trigger an automatic reactivation of its associated location, potentially explaining the long-term memory advantage observed in the *non-prioritization + testing* condition. This analysis was feasible because, during the working memory phase, each object appeared in four different trials. We categorized the objects into three groups based on the frequency of serving as a probe: zero times, once, or twice. Importantly, due to the low number of trials, we could not include serving as a probe three or four times in the analysis.

**Fig. 5:**
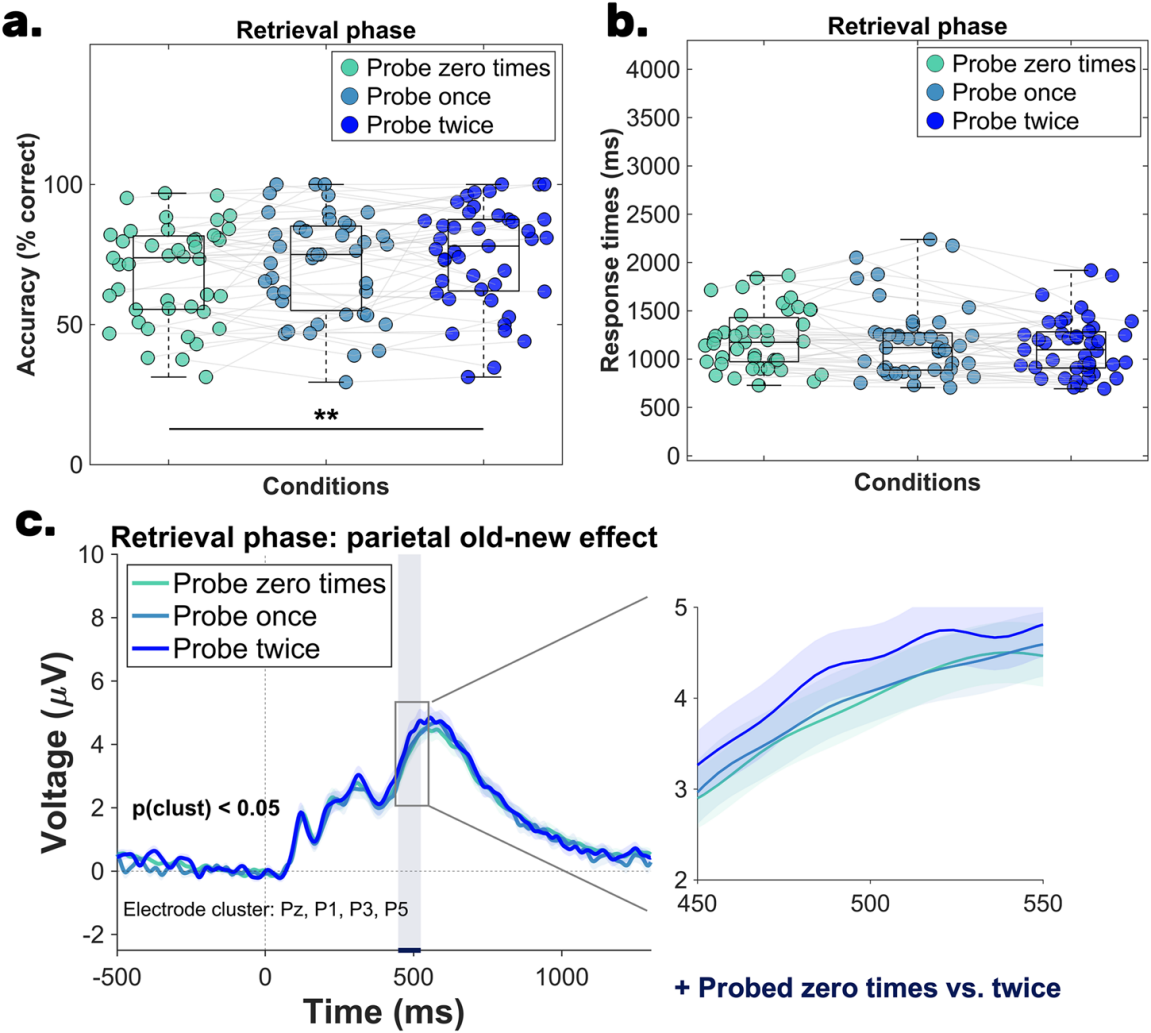
Results relative to the *non-prioritization + testing* condition during the final retrieval phase (*N* = 43). **a** Mean accuracy across three probing conditions: served as the central *probe zero times, once, and twice*. The central mark in each boxplot indicates the median, with the bottom and top edges representing the 25th and 75th percentiles, respectively. Gray lines connect data points from the same participant. Statistical significance is marked with *** for *p* < 0.001. **b** Mean response times for the same conditions, following the same boxplot conventions. **c** Illustrates the parietal old-new effect during retrieval, recorded at electrode sites Pz, P1, P3, and P5. Shaded areas around each ERP curve indicate the standard error of the mean. A significant cluster-based permutation effect (*p* < 0.05) was observed for the contrast between a probe zero times vs. twice (448-524 ms).

For the accuracy comparison, we conducted a rm-ANOVA (factors: presented as the central probe zero times, once, and twice), which revealed a main effect of probing, *F*(2, 84) = 6.48, *p* = 0.002, η_p_^2^ = 0.13, *BF*_*10*_ = 13.08, with the Bayesian analysis suggesting substantial evidence in favor of the alternative hypothesis. A comparable result was also revealed by the Friedman test, χ²(42) = 9.62, *p* = 0.008, Kendall’s W = 0.11. Our descriptive analysis (see Table 6) suggests that participants were the most accurate when the object served twice as the central probe, followed by objects that were central probes only once during the working memory task. The lowest accuracy was obtained for objects that never served as the central probe. The post-hoc analyses revealed a significant effect and strong evidence in favor of the difference between serving as a probe *zero times* vs *twice*, *t*(42)  =  −3.85, *p*_adj_  = 0.001, *d*_av_  =  0.33, 95% CI [−9.10, −2.84], *BF*_*10*_ = 68.68 and *W* = 195, *z* = −3.35, *p*_adj_  =  0.002, *r* = −0.51. The lack of accuracy difference between serving as a probe *zero times* vs *once* was supported by weak evidence, *t*(42)  = −1.54, *p*_adj_  = 0.12, *d*_av_  =  0.14, 95% CI [−6.12, 0.81], *BF*_*01*_ = 2.01 and and *W* = 326, *z* = −1.56, *p*_adj_ = 0.11, *r* = −0.23. Similarly, the lack of accuracy difference between serving as a probe *once* vs *twice* was supported by weak evidence, *t*(42)  =  −1.93, *p*_adj_ = 0.08, *d*_av_  = 0.18, 95% CI [−6.77, 0.13], *BF*_*01*_ = 1.10 and *W* = 299.50, *z* = −1.69, *p*_*ad*__*j*_ = 11, *r* = −0.25 (see Fig. 5a).

**Table 6:** Descriptive statistics

Mean and standard deviation					
Probing number	*N*	Mean accuracy	SD accuracy	Mean response times	SD response times
Zero times	43	68.70%	17.34%	1198.18 ms	304.45 ms
Once	43	71.36%	18.44%	1186.83 ms	383.72 ms
Twice	43	74.68%	18.22%	1123.57 ms	296.96 ms

The same rm-ANOVA was also conducted for response times. Notably, all trials were included in the analysis regardless of accuracy. No main effect was found, *F*(2, 84) = 2.63, *p* = 0.07, η_p_^2^ = 0.05, *BF*_*01*_ = 1.56, and the Bayes factor indicated only weak evidence in favor of the null hypothesis (see Fig. 5b). This pattern was also supported by the Friedman test, χ²(42) = 4.79, *p* = 0.09, Kendall’s W = 0.05.

The parietal old-new effect mirrors the accuracy results. The statistical comparison between serving as a probe *zero times* vs *twice* showed a significant cluster between 448 ms-524 ms. However, we did not find a statistically reliable effect for serving as a probe *zero times vs once* and *once* vs *twice* contrasts (Fig. 5c).

Overall, these results suggest that the number of times participants encounter the object as the central probe during the working memory task impacts long-term memory retrieval. This might be due to the automatic location reactivation occurring each time participants are presented with a certain object as a central probe during the working memory task.

### Comparable location representations across phases - decoding evidence from Experiment 2

Given that both experiments indicated that processing in working memory enhances long-term memory retrieval, we sought to further explore how location information is retrieved and how its neural representation might evolve throughout the phases. We trained a linear classifier to distinguish neural patterns associated with one of the four spatial locations participants had learned to associate with each object. Importantly, the decoding analysis was applied across phases: the classifier was trained and tested on all combinations of phases: encoding-working memory phase, encoding-retrieval phase, and working memory-retrieval phase. Importantly, successful cross-task decoding would suggest that the representational format of the location information reactivated in the working memory task and during the retrieval phase is highly comparable.

**Fig. 6:**
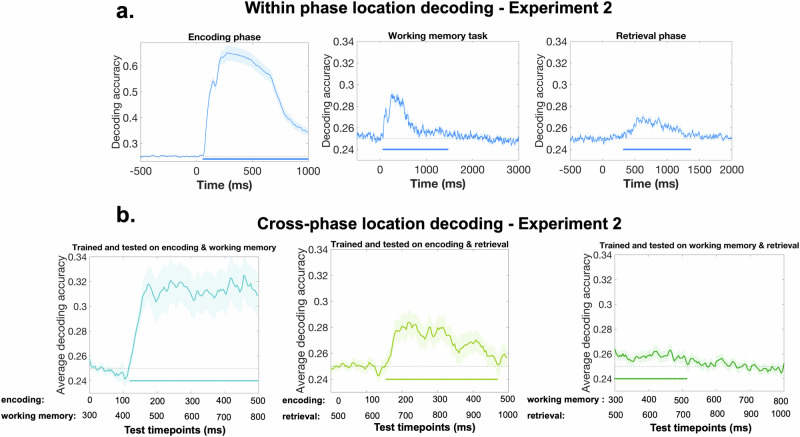
Decoding results (*N* = 43). **a** Within-phase decoding accuracy for the encoding phase (left), working memory task (middle), and retrieval phase (right). Decoding accuracy is plotted over time relative to stimulus onset, with shaded areas indicating the standard error of the mean. Solid horizontal lines mark significant clusters where accuracy exceeded chance (0.25, given four possible locations), as determined by a cluster-based permutation test: encoding phase (60-996 ms), working memory phase (68-1460 ms), and retrieval phase (328-1360 ms). **b** Cross-phase decoding accuracy for three phase combinations: encoding–working memory (left), encoding–retrieval (middle), and working memory–retrieval (right). In each case, data from both phases served as training and testing sets in a reciprocal fashion. For example, in the left panel, decoding involved training on encoding-phase data (0-500 ms) and testing on working memory data (300-800 ms), and vice versa; the plotted accuracy reflects the average across both directions. Accuracy is shown as a function of testing time. Each decoding analysis used a 500 ms time window specific to the corresponding phase: encoding (0-500 ms), working memory (300-800 ms), and retrieval (500-1000 ms). Shaded regions represent the standard error of the mean; horizontal lines indicate significant clusters where decoding performance was above chance. Our analysis revealed the following significant clusters: (i) encoding-working memory: 120-496 ms/420-796 ms; encoding-retrieval: 156-468 ms/656-968 ms; working memory-retrieval: 300-512 ms/500-712 ms.

Before conducting cross-phase decoding, we performed a within-phase analysis to confirm that location decoding was possible within each phase. As shown in Fig. 4a, decoding was indeed successful. Statistical analyses revealed the following significant time windows: 60-996 ms for the encoding phase, 68-1460 ms for the working memory task, and 328-1360 ms for the final retrieval phase (Fig. 6a).

Next, we performed the cross-phase decoding analyses, using EEG data from specific time windows selected based on the task dynamics. For the encoding phase, we focused on the 0–500 ms window to capture neural activity during object presentation. For the working memory task, the 300–800 ms window was chosen to reflect the maintenance period. Finally, for the retrieval phase, data from the 500–1000 ms window was analyzed, as prior research suggests this timeframe corresponds to memory retrieval processes^44^.

As illustrated in Fig. 6b, all cross-phase decoding analyses were successful. When the model was constructed using data from the encoding and working memory phases, a significant decoding window emerged between 120-496 ms/420-796 ms (Fig. 6b, left). Similar results were obtained for the encoding and retrieval phases, revealing a significant cluster between 156-468 ms/656-986 ms (Fig. 6b, middle). Finally, a significant cluster between 300-512 ms/500-712 ms (Fig. 6b, right) was observed when training and testing were conducted on the working memory and retrieval phases. Importantly, we conducted an additional control analysis, in which we corrected for eye movements by removing the ICs labeled by the ICLabel plug-in as eye components. Our results showed that the same pattern holds even after this correction is performed, suggesting that eye movements do not explain the decoding results (see Supplementary Materials, Note 4).

## Discussion

In two experiments, we investigated whether attentional prioritization and testing in working memory enhance long-term memory retrieval. Additionally, we explored the neural mechanisms underlying this enhancement. We obtained three key findings. First, both attentional prioritization and testing in working memory led to improved long-term memory performance, accompanied by enhanced neural representations compared to the control condition (i.e., items absent from working memory). Second, we found that repeatedly serving as the central probe in the working memory task provided additional long-term memory benefits. Specifically, objects that appeared twice as central probes yielded the highest retrieval accuracy—outperforming those that appeared only once or not at all. Notably, this effect emerged despite the absence of a location report during the working memory test. Third, our cross-task decoding analyses revealed that classifiers trained on neural data from any phase could successfully generalize to other phases. This suggests that a likely neural mechanism underlying the observed long-term memory enhancements is the repeated reactivation of the location representation each time the same object is re-encountered across phases. We interpret these findings within the framework of existing memory theories, highlighting the dynamic role of working memory in shaping long-term memory representations.

### Attentional prioritization and testing in working memory boost long-term memory retrieval

A key finding of the current experiment, supported by both behavioral and neural evidence, is that attentional prioritization within working memory enhances long-term memory retrieval compared to the control condition. These results align with prior research indicating that spatial retro-cues, which direct attention within working memory, boost the representation and lead to increased behavioral performance^13,49^. The current study suggests that this boost goes beyond the working memory domain and its effect extends to long-term memory. Additionally, these findings complement earlier research showing that different processes in working memory (i.e., elaboration, attentional prioritization, and testing) can lead to a differential build-up of information in long-term memory^9–14,16–25,50^. However, the present study offers a new perspective in this area, because it reveals that even already formed long-term memories benefit from processing in working memory.

Another key finding of this study is that testing in working memory also enhances subsequent long-term memory retrieval compared to the control condition, as evidenced by both behavioral and neural data. Notably, this enhancement occurs even though location information becomes irrelevant for the working memory test once the neutral cue is presented. To respond during the working memory probe, participants only need to maintain the objects’ identity and compare it to the centrally presented probe item.

Regarding the comparison between the benefits of attentional prioritization and testing, behavioral results showed that attentional prioritization provided a greater advantage. However, this was not reflected at the neural level. This discrepancy may be due to the short-lived neural effects of attentional prioritization, which EEG might not detect at the scalp level. Therefore, more sensitive imaging methods, like fMRI or intracranial EEG, may be needed to capture these subtle neural changes.

### Serving repeatedly as the central probe in working memory leads to an additional long-term memory retrieval benefit

To better understand the source of long-term memory retrieval enhancement in the *non-prioritization + testing* condition, we conducted a follow-up behavioral and EEG analysis. Here, we compared the final long-term memory retrieval performance and brain activity for objects serving as a probe zero, one, or two times in the working memory task. Results showed that objects presented twice as the memory probe led to higher long-term memory accuracy compared to objects never used as probes, with intermediate accuracy for objects presented once as a probe. This pattern was mirrored in the EEG data, where we found a difference in the amplitude of the parietal old-new component between the tested zero times vs twice conditions. Taken together, these findings suggest that serving as a probe in working memory functions as a complementary mechanism - beyond attentional prioritization and testing-that supports long-term memory retrieval.

A plausible interpretation of the long-term memory benefit in this context likely stems from a combination of involuntary and voluntary processes engaged during the working memory probe presentation. First, each time the previously encoded working memory object reappears as a probe, its associated location representation may be involuntarily reactivated—even though the location information is not relevant for the working memory test. This automatic reinstatement could help reinforce object-location bindings. Second, on a more deliberate level, the act of evaluating whether the probe matches any item in working memory requires participants to re-access, manipulate, and compare stored representations. This process goes beyond passive recognition, engaging additional cognitive operations that may strengthen the location-object association. Finally, repeated probe encounters may amplify the combined impact of these voluntary and involuntary mechanisms, leading to a cumulative enhancement of the object-location association.

### Evidence for a shared representational format of location information across phases

To further investigate the neural mechanisms underlying the retrieval and evolution of  location representations,  we employed a pairwise cross-phase decoding approach. Using this method, we trained and tested a linear classifier across all combinations of task phases. Notably, classification accuracy remained reliably above chance for all phase pairs, including encoding-working memory, working memory-retrieval, and encoding-retrieval. The consistent above-chance decoding accuracy suggested a stable and highly similar neural representation of location information throughout the three phases of the task. These findings align with the other results and support the idea that, across repeated encounters with the same object, the neural patterns underlying the retrieved location information are reinstated with high fidelity. Such reinstatement may facilitate long-term memory enhancement by maintaining a consistent representational format for the object’s location across time.

With respect to existing memory theories, this pattern is compatible with two major frameworks: one positing the strengthening of a single memory trace through repeated reactivation, and another proposing the formation of multiple, redundant traces. According to the first view, the classifier’s success reflects the repeated engagement of the same underlying trace, which becomes more robust with each retrieval or reactivation. In contrast, the multiple-trace account would suggest that each re-encounter generates a new, distinct trace—but one that represents the location information in a similar neural format. In this case, the classifier can generalize across phases because it detects recurring representational features common to these separate, yet highly similar, memory traces. Future research will be needed to disentangle these two possibilities and determine whether long-term memory benefits arise primarily from a single trace reinforcement, multiple trace formation, or a combination of both.

### Limitations

#### Interpretational boundaries

As suggested in the previous section, our findings suggest that repeated reactivation of location representations supports long-term memory enhancement. Nevertheless, the current study does not definitively resolve whether this reflects the strengthening of a single memory trace or the creation of multiple traces. Future studies designed to selectively manipulate encoding-retrieval similarity could help adjudicate between these theoretical perspectives.

#### Sample representativeness

Our sample was composed primarily of young, highly educated individuals recruited through convenience sampling. While this approach is common and well-suited for controlled experimental designs, it naturally limits the generalizability of the findings to the broader population. Specifically, it remains an open question whether the observed effects hold across more diverse demographic groups, including older adults or individuals with varying educational backgrounds. Future studies employing more representative samples will be essential to assess the broader applicability of these mechanisms. Nonetheless, the current results offer a robust foundation for understanding memory dynamics within a well-characterized population and provide a valuable starting point for further exploration in more diverse cohorts.

## Conclusions

To summarize, our study provides insights into the interplay between working memory and long-term memory. Across two experiments involving 86 participants, we manipulated attentional prioritization and testing in working memory to examine their effects on the retrieval of newly formed long-term memory representations. Our findings reveal three key contributions. First, both attentional prioritization and testing in working memory boosted long-term memory performance and its corresponding neural representation compared to a control condition (i.e., items not presented in the working memory task). Second, serving as a probe in working memory emerged as a distinct factor contributing to long-term memory enhancement, with benefits observed at both behavioral and neural levels. Third, neural data showed that repeated encounters with the same object led to the reinstatement of location-specific neural patterns, potentially serving as a mechanism underlying the observed long-term memory boost. Our findings extend existing theoretical views by suggesting that working memory does more than temporarily store retrieved long-term memory information; it can actively strengthen it. This suggests that the relationship between the two memory systems is far more dynamic than previously thought, with working memory playing an active role in shaping long-term memory representations.

## Supplementary information

Transparent Review file

Supplementary Materials

Reporting summary

## Data Availability

The raw behavioral and EEG data are publicly available on Dryad: 10.5061/dryad.mgqnk99br.

## Code availability

All scripts used for the reported analyses are publicly available on Open Science Framework (OSF): https://osf.io/ne9yt/.
