# Supplementary material for: Processing in working memory boosts long-term memory representations and their retrieval
Source: Commun Psychol. 2025 Aug 26;3:129. doi: 10.1038/s44271-025-00309-3 (PMC12381095; doi:10.1038/s44271-025-00309-3)
Supplement: Supplementary file 2 — Supplementary Materials [file 44271_2025_309_MOESM2_ESM.pdf]

Supplementary materials

Processing in working memory boosts long-term memory representations and their retrieval

Melinda Sabo and Daniel Schneider

Supplementary notes 1: Technical details – Experiment 1 and 2

Table S1

Technical parameters – Experiment 1 and 2

Experiment	Parameter name	Parameter value
Experiment 1	Size of the intact object in all phases	253×177px or 177×253px, corresponding to 4.8°×3.35° or 3.35°×4.8° visual angle
Experiment 2	Size of the intact object in all phases	200 px×200 px, corresponding to a visual angle of 3.8°×3.8°
Experiment 1 and 2	Size of the scrambled objects in phase 2	162×162px, corresponding to 3.07°×3.07° visual angle
Experiment 1 and 2	Background color in all phases	RGB 128-128-128
Experiment 1 and 2	Size of the fixation dot in all phases	0.2°×0.2° visual angle
Experiment 1 and 2	Distance between the objects central and the fixation dot in all phases	~3° visual angle
Experiment 1 and 2	Selective retro-cue size	1°×0.5° or 0.5°×1° visual angle
Experiment 2	Neutral retro-cue size	1°×1° visual angle

The scrambled objects were created using the GIMP software (https://www.gimp.org/) with the fx_taquin command from the G´MIC plugin (https://gmic.eu/), resized to 162x162px before scrambling.

Supplementary notes 2: Higher accuracy in the *prioritization+testing* vs. *non-prioritization+testing* conditions when the number of probing instances is constant

As a control analysis, we examined whether the observed accuracy differences between the prioritization and testing conditions held when the number of probing instances was kept constant. We compared accuracy between these conditions when objects were probed once and when they were probed twice. For the probed-once condition, our analysis revealed substantial evidence for a difference between the prioritization and testing conditions, $t(42) = 3.23$, $p = .002$, $d_{av} = 0.34$, 95% CI [2.29, 9.93], $BF_{10} = 13.70$. Similarly, when objects were probed twice, the accuracy difference was supported by strong evidence, $t(42) = 4.03$, $p < .001$, $d_{av} = 0.30$, 95% CI [2.61, 7.85], $BF_{10} = 113.83$ (see descriptive statistics in Table S2).

Table S2
Descriptive statistics

Mean and standard deviation

Probing number	Condition	N	Mean accuracy	SD accuracy
Once	Prioritization	43	77.47%	16.93%
	Testing	43	71.36%	18.44%
Twice	Prioritization	43	79.91%	16.28%
	Testing	43	74.68%	18.22%

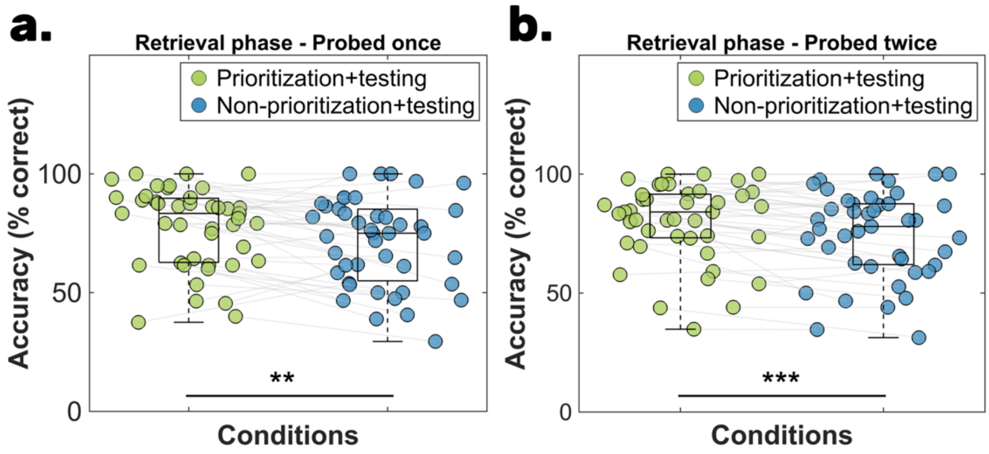

Figure S1. Panel a illustrates the mean accuracy in the prioritization and testing conditions for objects that served as a probe once during the working memory task, while panel b shows the accuracy difference for objects that served as a probe twice (N=43). The central mark in each boxplot represents the median, with the bottom and top edges indicating the 25th and 75th percentiles, respectively. The whiskers do not extend to averages, which are treated as outliers. Grey lines connect data points from the same participant. Statistical significance is indicated with the following symbols: * $p < 0.05$; ** $p < 0.01$; *** $p < 0.001$.

Supplementary notes 3: Comparable behavioral performance between Experiment 1 and 2

Given the higher number of objects to be learned in Experiment 2, we conducted an additional control analysis to ensure that behavioral performance in the final retrieval phase was comparable between experiments. To do this, we calculated the average accuracy and response times, then contrasted these measures using independent-sample t-tests (see Table S2). The results indicated no significant differences in accuracy, with evidence for the lack of difference, $t(42) = 0.60$, $p = .54$, $d_{av} = 0.12$, 95% CI [-0.04, 0.08], $BF_{01} = 5.08$. A similar pattern was obtained for response times, $t(42) = -0.47$, $p = .63$, $d_{av} = 0.10$, 95% CI [-173.70, 107.72], $BF_{01} = 5.45$.

Table S3

Descriptive statistics – condition-independent behavioral performance of Experiment 1 and 2

Mean and standard deviation

Experiment	N	Mean accuracy	SD accuracy	Mean response times	SD response times
Experiment 1	43	72.90%	14.89%	1144.61 ms	321.12 ms
Experiment 2	43	70.99%	15.92%	1177.60 ms	300.90 ms

Supplementary notes 4: Eye movements do not explain the decoding results

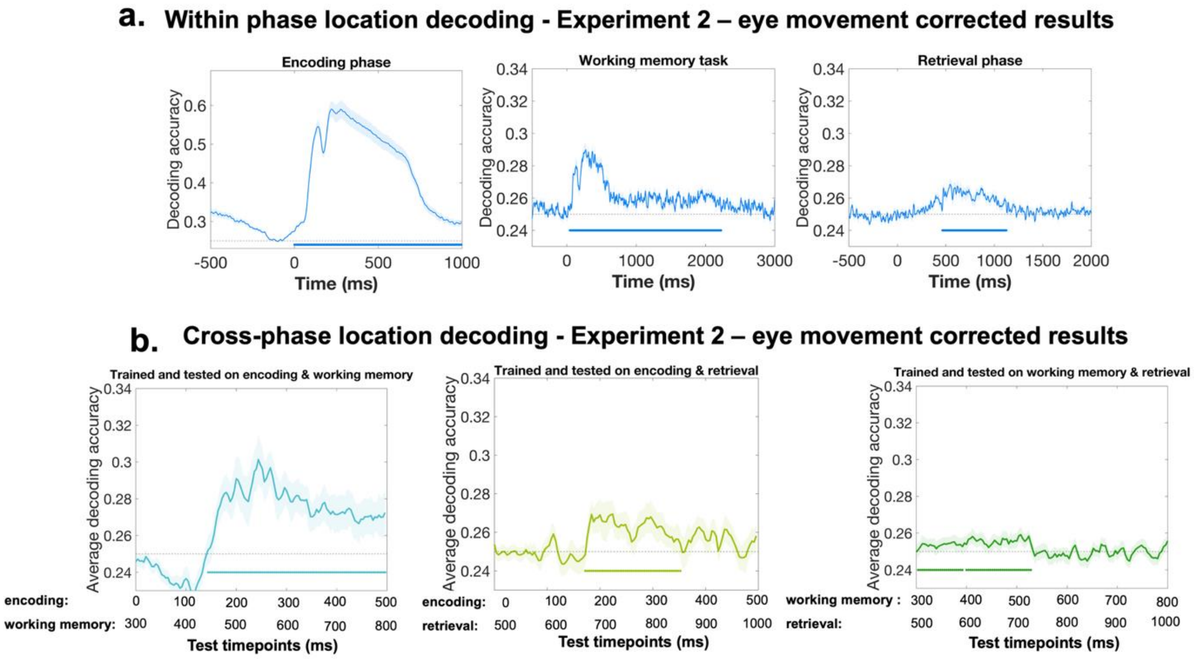

Figure S2. Decoding results after correcting for ocular artifacts (N=43). (a) Within-phase decoding accuracy for the encoding phase (left), working memory task (middle), and retrieval phase (right). Decoding accuracy is plotted over time relative to stimulus onset, with shaded areas indicating the standard error of the mean. Solid horizontal lines mark significant clusters where accuracy exceeded chance (0.25, given four possible locations), as determined by a cluster-based permutation test: encoding phase (60–996 ms), working memory phase (68–1460 ms), and retrieval phase (368–1420 ms). (b) Cross-phase decoding accuracy for three phase combinations: encoding–working memory (left), encoding–retrieval (middle), and working memory–retrieval (right). In each case, data from both phases served as training and testing sets in a reciprocal fashion. For example, in the left panel, decoding involved training on encoding-phase data (0–500 ms) and testing on working memory data (300–800 ms), and vice versa; the plotted accuracy reflects the average across both directions. Accuracy is shown as a function of testing time. Each decoding analysis used a 500 ms time window specific to the corresponding phase: encoding (0–500 ms), working memory (300–800 ms), and retrieval (500–1000 ms). Shaded regions represent the standard error of the mean; horizontal lines indicate significant clusters where decoding performance was above chance.

Given that previous research has identified eye movements as a potential confound in decoding analyses, we conducted an additional set of decoding analyses designed to minimize their influence. To this end, we incorporated independent component analysis (ICA) into our preprocessing pipeline to identify and remove components associated with ocular artifacts. Specifically, we applied a 1 Hz high-pass filter (as preparation for ICA) and used the ICLabel plug-in to identify components with more than 60% eye-related activity, which were then removed. All other preprocessing steps remained unchanged.

As shown in Figure S2, the decoding results remained robust after this ocular artifact correction. The key significant time windows observed were as follows: (i) within phase decoding, encoding phase: 0-996 ms; (ii) within phase decoding, working memory phase: 44-2224 ms; (iii) within phase decoding, retrieval phase: 464-1124 ms; (iv) cross-task decoding, encoding-working memory: 144-496 ms / 444-796 ms; (v) cross-task decoding, encoding-retrieval: 172-352 ms / 672-852 ms; (vi) cross-task decoding, working memory-retrieval: 304-428 ms / 504-728 ms.

Supplementary notes 5: Event-related potential results are replicable also when applies to an extended electrode cluster

To show that the event-related potential results are robust, we extended the electrode cluster to POz, Pz, CPz, PO3, PO4, PO7, PO8, PO9, PO10, P1, P2, P3, P4, P5, P6, P7, P8, P9, P10, CP1, CP2, CP3, CP4 and conducted the same analyses reported in the main text of the manuscript.

Results of this extended analysis replicate the results we reported in the manuscript both for Experiment 1 and 2. Specifically, the following significant clusters have been found for Experiment 1: (i) Prioritization+testing vs. non-prioritization: 492-600 ms; (ii) Prioritization+testing vs. absent in working memory: 244-604 ms and for Experiment 2: (i) Prioritization+testing vs. absent in working memory: 220-624 ms; (ii) Non-prioritization+testing vs. absent in working memory: 308-616 ms; (iii) Prioritization+testing vs. non-prioritization: 460-620 ms..

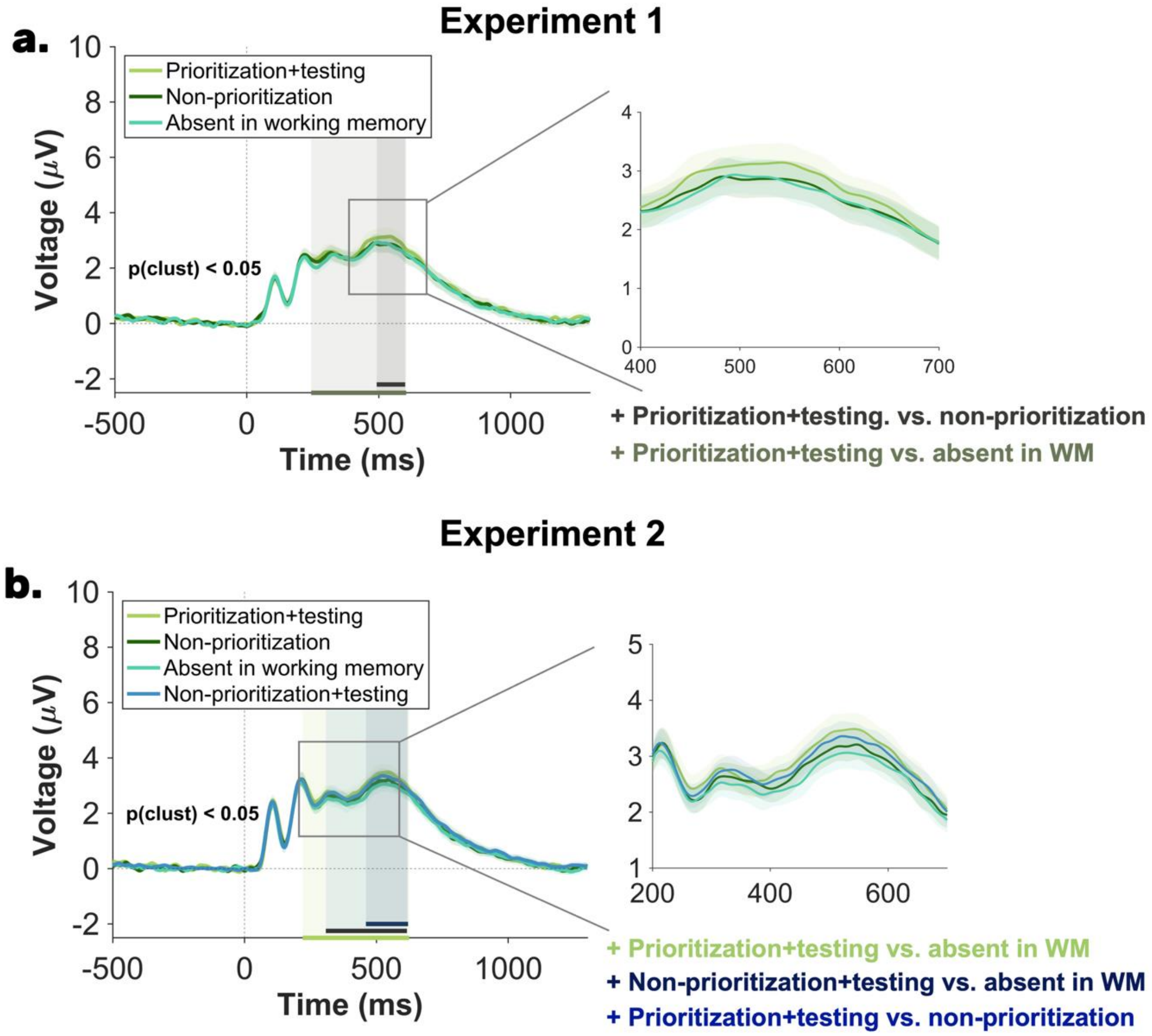

Figure S3. Replication of the event-related potential results on an extended electrode cluster (N=43). The shaded regions indicate the significant time window identified through the cluster-based permutation analysis. The shaded areas around the ERP of each condition denote the standard error of the mean.

Supplementary notes 6: Post-study questionnaire

After completing the main task, participants filled out a post-study questionnaire. They were asked whether they were aware that the same associations were used in the working memory phase as during the initial encoding phase (original German question: *War Ihnen bewusst, dass die zweite Phase auf den Assoziationen der ersten Phase aufbaute?"*). Subsequently, they were then asked whether they found this helpful (original German question: *In der zweiten Phase auf einige der Objekt-Positions-Assoziationen aus der ersten Phase zu stoßen, war:*).

For the first question, participants could respond with "yes" or "no." For the second, they could choose from the following four options: (i) useful (*nützlich*), (ii) confusing (*verwirrend*), (iii) neither useful nor confusing (*weder nützlich noch verwirrend*), (iv) I was not aware that the second phase was related to the first (*mir war nicht klar, dass die zweite Phase etwas mit der ersten Phase zu tun hat*). In response to the first question, 72.09% answered "yes," 23.25% answered "no," and 4.65% did not respond. For the second question, 59.09% of the participants found the repetition useful, 6.81% found it confusing, 25% indicated it was neither useful nor confusing, and 6.81% reported being unaware of the connection between the two phases. Overall, these results suggest that most participants recognized the connection between the encoding phase and the working memory task, and that more than half found the repetition useful—indicating an awareness that the objects reappeared on the same locations.

Supplementary notes 7: Long-term memory retrieval accuracy is comparable during the first and the second report

To increase the number of trials per condition, necessary for an adequate signal-to-noise ratio for the EEG analyses, items were tested twice during the final long-term memory retrieval phase. To illustrate that both repetitions can be treated equivalently, we compared participants accuracy across the two tests. Accuracy was highly consistent, which was supported by substantial evidence for the lack of difference in accuracy: (i) experiment 1: $t(42) = -0.64$, p

= .52, 95% CI [-0.01, 0.006], BF_{01} = 4.97; (ii) experiment 2: $t(42) = -0.42$, $p = .67$, 95% CI [-0.01, 0.008], BF_{01} = 5.57.
