# Supplementary material for: Processing in working memory boosts long-term memory representations and their retrieval
Source: Commun Psychol. 2025 Aug 26;3:129. doi: 10.1038/s44271-025-00309-3 (PMC12381095; doi:10.1038/s44271-025-00309-3)
Supplement: Supplementary file 3 — Reporting summary [file 44271_2025_309_MOESM3_ESM.pdf]

nature portfolio

Corresponding author(s): Melinda Sabo

Last updated by author(s): 6/18/2025

Reporting Summary

Nature Portfolio wishes to improve the reproducibility of the work that we publish. This form provides structure for consistency and transparency in reporting. For further information on Nature Portfolio policies, see our Editorial Policies and the Editorial Policy Checklist.

Statistics

For all statistical analyses, confirm that the following items are present in the figure legend, table legend, main text, or Methods section.

n/a	Confirmed	
☐	☒	The exact sample size (*n*) for each experimental group/condition, given as a discrete number and unit of measurement
☐	☒	A statement on whether measurements were taken from distinct samples or whether the same sample was measured repeatedly
☐	☒	The statistical test(s) used AND whether they are one- or two-sided<br>*Only common tests should be described solely by name; describe more complex techniques in the Methods section.*
☒	☐	A description of all covariates tested
☐	☒	A description of any assumptions or corrections, such as tests of normality and adjustment for multiple comparisons
☐	☒	A full description of the statistical parameters including central tendency (e.g. means) or other basic estimates (e.g. regression coefficient) AND variation (e.g. standard deviation) or associated estimates of uncertainty (e.g. confidence intervals)
☐	☒	For null hypothesis testing, the test statistic (e.g. *F*, *t*, *r*) with confidence intervals, effect sizes, degrees of freedom and *P* value noted<br>*Give P values as exact values whenever suitable.*
☐	☒	For Bayesian analysis, information on the choice of priors and Markov chain Monte Carlo settings
☒	☐	For hierarchical and complex designs, identification of the appropriate level for tests and full reporting of outcomes
☐	☒	Estimates of effect sizes (e.g. Cohen's *d*, Pearson's *r*), indicating how they were calculated

Our web collection on statistics for biologists contains articles on many of the points above.

Software and code

Policy information about availability of computer code

Data collection	Stimuli were presented using a ViSaGe MKII Stimulus Generator (Cambridge Research Systems, UK). The experiment was programmed in Lazarus IDE (Free Pascal).
Data analysis	All data analyses were conducted using MATLAB v2023b. Custom-written scripts were used, incorporating functions from the EEGLAB toolbox (v14.1.2.b; Delorme and Makeig, 2004) and the CoSMoMVPA toolbox (Oosterhof et al., 2016).

For manuscripts utilizing custom algorithms or software that are central to the research but not yet described in published literature, software must be made available to editors and reviewers. We strongly encourage code deposition in a community repository (e.g. GitHub). See the Nature Portfolio guidelines for submitting code & software for further information.

Data

Policy information about availability of data

All manuscripts must include a data availability statement. This statement should provide the following information, where applicable:

- Accession codes, unique identifiers, or web links for publicly available datasets
- A description of any restrictions on data availability
- For clinical datasets or third party data, please ensure that the statement adheres to our policy

The raw data and all the scripts used for the reported analyses will be publicly available after publication on Open Science Framework (OSF): https://osf.io/ne9yt/

Research involving human participants, their data, or biological material

Policy information about studies with human participants or human data. See also policy information about sex, gender (identity/presentation), and sexual orientation and race, ethnicity and racism.

Reporting on sex and gender	Participants self-reported their sex in a demographic questionnaire completed at the beginning of the experiment. We aimed for a relatively balanced sample, with both Experiment 1 and Experiment 2 including 25 females and 18 males.
Reporting on race, ethnicity, or other socially relevant groupings	No data on participants' race or ethnicity were collected.
Population characteristics	See above.
Recruitment	Participants were healthy young adults (age range: 18–35 years) recruited from the Rhein-Ruhr area in Germany via online platforms (Facebook, Moodle), including those affiliated with local universities. As a result, the sample primarily consisted of students or recent graduates. Given that most participants had completed higher education, our findings may not generalize to the broader population, particularly individuals with different educational backgrounds.
Ethics oversight	The study was approved by the Ethics Committee of the Leibniz Research Centre for Working Environment and Human Factors (Dortmund, Germany) and conducted in accordance with the Declaration of Helsinki.

Note that full information on the approval of the study protocol must also be provided in the manuscript.

Field-specific reporting

Please select the one below that is the best fit for your research. If you are not sure, read the appropriate sections before making your selection.

☐ Life sciences ☒ Behavioural & social sciences ☐ Ecological, evolutionary & environmental sciences

For a reference copy of the document with all sections, see nature.com/documents/nr-reporting-summary-flat.pdf

Behavioural & social sciences study design

All studies must disclose on these points even when the disclosure is negative.

Study description	This study comprises two experiments using a quantitative experimental design to collect neural and EEG data.
Research sample	The research sample consisted of healthy young adults (age range: 18–35 years) from the Rhein-Ruhr area in Germany. The average age was 23.65 years in Experiment 1 and 24.97 years in Experiment 2. Both experiments included 25 women and 18 men. Given that most participants held a bachelor's or master's degree, the sample is not fully representative of the general population.
Sampling strategy	A convenience sampling approach was used. Study advertisements were posted on various online platforms, and participants who met the eligibility criteria voluntarily signed up. Sample size was determined based on prior long-term memory studies conducted in our lab (Sabo et al., 2022), which balances statistical power and cost efficiency.
Data collection	Participants completed a demographic questionnaire at the beginning of the study and a follow-up questionnaire after the main experiment, both in paper-and-pencil format. The main experiment was conducted on a computer, with EEG recordings of participants' brain activity. Data collection was primarily conducted by research assistants (with some exceptions) who were blind to the study's hypotheses. The first author of the manuscript was present for approximately 20% of data collection sessions in Experiment 1 and 50% in Experiment 2.
Timing	Experiment 1: Data collection began on December 16, 2022, and concluded on April 14, 2023.<br>Experiment 2: Data collection began on August 9, 2023, and concluded on December 14, 2023.<br>Data collection was continuous within these time frames, with data collected each month.
Data exclusions	Experiment 1: Six datasets were excluded—four due to EEG recording failures resulting in data loss, and two due to task misunderstandings (one participant consistently pressed the right button during the final retrieval phase, the second one was excluded due to failure in understanding the task instructions, as documented by the research team during data collection).<br>Experiment 2: Two datasets were excluded—one for performing at chance level (25%) and another for reaction times exceeding six standard deviations from the group mean.
Non-participation	No participants dropped out or declined participation.
Randomization	Each participant completed all experimental conditions.

Reporting for specific materials, systems and methods

We require information from authors about some types of materials, experimental systems and methods used in many studies. Here, indicate whether each material, system or method listed is relevant to your study. If you are not sure if a list item applies to your research, read the appropriate section before selecting a response.

Materials & experimental systems

n/a	Involved in the study
☒	☐ Antibodies
☒	☐ Eukaryotic cell lines
☒	☐ Palaeontology and archaeology
☒	☐ Animals and other organisms
☒	☐ Clinical data
☒	☐ Dual use research of concern
☒	☐ Plants

Methods

n/a	Involved in the study
☒	☐ ChIP-seq
☒	☐ Flow cytometry
☒	☐ MRI-based neuroimaging

Plants

Seed stocks	not applicable
Novel plant genotypes	not applicable
Authentication	not applicable
