# Supplementary material for: Processing in working memory boosts long-term memory representations and their retrieval
Source: Commun Psychol. 2025 Aug 26;3:129. doi: 10.1038/s44271-025-00309-3 (PMC12381095; doi:10.1038/s44271-025-00309-3)
Supplement: Supplementary file 1 — Transparent Review file [file 44271_2025_309_MOESM1_ESM.pdf]

nature portfolio

Peer Review File

Processing in working memory boosts long-term memory representations and their retrieval

Corresponding Author: Dr Melinda Sabo

This file contains all editorial decision letters in order by version, followed by all author rebuttals in order by version.

Version 0:

Decision Letter:

Dear Dr Sabo,

Thank you for submitting your manuscript titled "Processing in working memory boosts long-term memory representations and their retrieval" and thank you for your patience in awaiting an initial decision. I have now had the opportunity to read your work and discuss it with the other editors in the Communications Psychology team. Regretfully, we cannot publish this submission in Communications Psychology.

It is our policy to decline a substantial proportion of manuscripts without sending them to referees so that they may be sent elsewhere without further delay. Such decisions are made by the editorial staff when it appears that papers do not meet the criteria for publication in Communications Psychology.

We appreciate your study, which demonstrates across two studies how attentional prioritization and intermittent testing contribute to improved memory performance in a later recall task.

At this stage, we consider whether the work represents an advance of significant relevance to scientists working in the same area of psychology; whether the data appear appropriately substantive and the analysis suitable to address the research question; and whether there is sufficient evidence in support of the conclusions.

Judging the work against the journal’s criteria for publication, I am sorry to say that in this case we are not persuaded that the work presents an advance of sufficient interest to other scientists in the field.

Please note that we do not question the validity of your work.

Although we cannot offer to publish your manuscript, I suggest that you consider npj Science of Learning as a suitable venue for this work. To transfer your manuscript, please use our manuscript transfer portal. You will not have to re-supply manuscript metadata and files, unless you wish to make modifications. For more information, please see our manuscript transfer FAQ page.

I am sorry that we cannot offer further consideration for publication in Communications Psychology and thank you for your interest in publishing with us.

Best regards,
Marike Schiffer

Marike Schiffer, PhD
Chief Editor
Communications Psychology

Version 1:

Decision Letter:

** Please ensure you delete the link to your author homepage in this e-mail if you wish to forward it to your coauthors **

Dear Dr Sabo,

Thank you for your patience during the peer-review process. Your manuscript titled "Processing in working memory boosts long-term memory representations and their retrieval" has now been seen by 2 reviewers, and I include their comments at the end of this message. They find your work of interest but raised some important points. We are interested in the possibility of publishing your study in Communications Psychology, but would like to consider your responses to these concerns and assess a revised manuscript before we make a final decision on publication.

We therefore invite you to revise and resubmit your manuscript, along with a point-by-point response to the reviewers. Please highlight all changes in the manuscript text file.

Editorially, we consider it critical that both reviewers' requests for additional analyses are met. We also ask that the presentation of the analysis is overall improved, and that any analysis choices (incl preprocessing decisions) are well justified.

We are committed to providing a fair and constructive peer-review process. Please don't hesitate to contact us if you wish to discuss the revision in more detail.

I am attaching an Editorial Requests Table that details critical reporting requirements for the revised manuscript. Please attend to each item and ensure your manuscript is fully compliant. If your revised manuscript is not aligned with these requests on major issues, such as those concerning statistics, it may be returned to you for further revisions without re-review.

Please submit the following items:

- Revised manuscript
- Point-by-point response to the referees' comments
- Cover letter (as a separate document)
- Nature Research Reporting Summary
- Editorial Policy Checklist
- Completed Editorial Request Table (attached).

via this link: Link Redacted .

** This url links to your confidential home page and associated information about manuscripts you may have submitted or be reviewing for us. If you wish to forward this email to co-authors, please delete the link to your homepage first **

Additional guidance is available in our style and formatting guide Communications Psychology formatting guide.

We hope to receive your revised paper within 8 weeks; please let us know if you aren't able to submit it within this time so that we can discuss how best to proceed. If we don't hear from you, and the revision process takes significantly longer, we may close your file. In this event, we will still be happy to reconsider your paper at a later date, provided it still presents a significant contribution to the literature at that stage.

We would appreciate it if you could keep us informed about an estimated timescale for resubmission, to facilitate our planning.

We look forward to seeing the revised manuscript and thank you for the opportunity to review your work.

Best regards,

Marike

Marike Schiffer, PhD

Chief Editor
Communications Psychology

REVIEWER EXPERTISE:
Reviewer #1: memory, cognitive neuroscience/neuroimaging
Reviewer #2: memory, cognitive neuroscience/neuroimaging

REVIEWER REPORTS:

Reviewer #1 (Remarks to the Author):

This study addresses an important and timely question - whether working memory can actively modify existing long-term memories. The authors employ a well-structured design in which adult participants learn object-location associations, engage in a working memory task involving attentional prioritization and testing, and later retrieve these associations from long-term memory. The behavioral findings are solid and convincingly demonstrate that both prioritization and testing improve long-term recall, with repeated probes further enhancing performance. The use of EEG combined with decoding analysis to examine shared memory traces across phases is a compelling approach. However, I have some reservations about the EEG-related findings. While the integration of ERP and classification methods is promising, aspects of the methodology are not clearly described, which limits interpretability.

Below, I detail specific issues that require clarification or revision:

Majors

1. Electrode Selection:
- It is unclear why EEG analyses are centered exclusively on three electrodes (P1, P3, and P5). While the LPC is typically observed over left-parietal sites, restricting analyses to only these electrodes may limit sensitivity to broader, potentially relevant activity patterns. A rationale for this choice, or an expanded analysis, would strengthen the conclusions.
- Additionally, there is an inconsistency between the methods and results sections: the methods specify analyses on P1, P3, and P5 (p. 28), yet the results and figures also include Pz. Please clarify which electrodes were analyzed and ensure consistency throughout the manuscript.

2. Decoding Feature Source:
The manuscript does not specify where MVPA features were extracted from. Were features limited to the same three electrodes used for ERP analyses? If so, this would significantly constrain the spatial richness MVPA can leverage. Clarification is needed on which channels were included in the classification analysis and how features were defined.

3. Eye Movement Artifacts:
Given the spatial arrangement of visual stimuli, eye movements are likely. However, MVPA analyses appear to include all trials without rejecting those possibly contaminated by ocular artifacts. This raises concerns about the validity of the neural signal. Artifact rejection procedures should be consistently applied across all EEG analyses, including MVPA.

4. Downsampling Discrepancy:
There is an inconsistency regarding the downsampling rate: some sections report downsampling to 200 Hz, while others state 250 Hz. This should be corrected and consistently reported throughout the manuscript.

5. Inconsistent Preprocessing Pipelines:
- The preprocessing steps vary across different analyses. For instance, what is considered an artifact in one analysis seems to be accepted as valid in another. A clearly defined and consistent preprocessing pipeline across analyses is essential.
- While the use of cluster-based permutation tests is well described for ERP analyses, the procedure for statistical evaluation in the decoding analysis is unclear. Did the authors permute category labels during training, testing, or both? Cluster-based permutation testing in classification contexts is not straightforward, and further detail is necessary for interpretation.

6. EEG preprocessing appears to be described in multiple places with varying details, which makes it difficult to follow. Below are specific points that need clarification:
- An artifact detection threshold of 500 µV is unusually high. Why not use the more standard 100 µV?
- The manuscript mentions that a maximum of 5% trial rejection is allowed (line 3, p. 27). What happens when this threshold is exceeded?
- The EEG recording setup is not described: What system was used? How many channels were recorded? What was the sampling rate? What were the online filters and referencing procedures?
- On page 26, Tables 1 and 2 are referenced in the EEG methods section, but these tables contain only behavioral statistics.
- The statement that “anterior channels were excluded from rejection” (p. 26) is unclear. Please specify which channels this refers to.
- The baseline period is defined as -200 to 0 ms, but the inter-trial interval during retrieval ranges from 100 to 1500 ms. This raises the possibility that some baselines may include motor-related activity from the previous trial, which should be addressed.

7. Interpretation of Cross-Phase Decoding:
While the above-chance cross-phase decoding is intriguing, the conclusion that it reflects memory strengthening or the generation of new traces is not fully supported. An important issue that could strengthen the interpretation would be to assess the extent to which the reactivation findings are trial-specific. However, even if reactivation were shown to be trial-specific, one might still expect that previous and novel memories would not necessarily build on entirely orthogonal representational formats. Although this is an important debate, I am not convinced that the current results provide a strong enough case to resolve this crucial issue in the field.

Minors
- The discussion section references results from a questionnaire, which appears to have been used to justify the exclusion of one participant. However, this questionnaire is not described anywhere in the methods section. Since data from this instrument were used to inform both the interpretation of results and participant exclusion, it is essential that the authors provide a full description of the questionnaire, including its content, how and when it was administered, and the analysis procedures applied. Additionally, the criteria used to exclude participants based on this questionnaire should be clearly stated to ensure transparency and reproducibility.

Reviewer #2 (Remarks to the Author):

Sabo and colleagues conducted two combined long-term + working memory experiments and recorded EEG in order to assess whether attentional prioritization and testing in working memory (WM) can boost long-term memory (LTM) performance and the associated neural mechanisms. In both experiments, participants studied object-location pairs, performed a WM task on a subset of the objects, and then performed a location test on the objects. The authors manipulated whether an object was: both cued and tested during the WM phase, not cued but tested during the WM phase, not cued and not tested (but presented) during the WM phase, or not presented during the WM phase. They find that cue+testing out performs the other conditions, including testing without cueing. They find elevated neural signatures of recollection for test probes that were cued and tested. Finally, they find evidence that location information from the study phase is recapitulated during both the WM and test phase. The findings suggest that automatic retrieval of stored information of items entered into WM facilitates later long term recollection.

I find the results of the paper interesting and compelling. The role of WM in LTM formation/retrieval is an active area of interest and I think that this work will make a valuable contribution. I do have some questions/concerns related to some of the conditions and interpretations, but I think that they are all addressable.

Major

1. Figure 1 is fantastic for laying out the conditions, but I think that there is an important condition that exists, but was not tested (perhaps due to small trial counts). Specifically, what is the impact on later memory (and the other outcome variables) for items that were prioritized and not tested? For instance, in the case where the selective cue is the horizontal line and the peacock is tested, what happens to memory for the doll? This could provide very interesting information since at the moment it's clear that the combination of prioritization and testing is better than testing alone, but the impact of prioritization alone is unclear.

2. I'm not convinced by the single vs. multi-trace account of how the location information is being represented and I don't think that this interpretation is critical to the paper. The cross-phase decoding indicates that at least some similarly coded location information is shared across phases, but does not rule out the possibility that each phase may additionally contain unique location information. Furthermore, I don't think that the emphasis on non-consolidated information is warranted, especially given that there is no comparison with presumed consolidated information. Also, a minor point, but it is not certain that an item presented during the study phase is encoded, thus the current study is not necessarily testing "encoded but not yet consolidated representations."

3. Cross-phase location decoding should fail when applied to the WM maintenance period in the neutral condition in E2, thus it would be beneficial to separate out cued from neutral trials in the analysis/figures. If WM enhances LTM through automatic reinstatement of location information during the WM maintenance phase, then the degree of location information during the WM maintenance phase should predict test performance. The authors should run a regression analysis to test this prediction which would further support their interpretations.

4. In a few locations (page 11/ line 16, page 13/line 4) the authors state that their results suggest "a benefit of attention selection over testing," but this could be read as "attention selection (alone) is better than testing (alone)" which is not accurate given that the comparison is between the combination of attention selection and testing vs. testing alone.

5. In the methods (Page 23 / Line 10) cues are described as being 100% valid but it is unclear how the cues could possibly be invalid. Given that the WM test prompt is "does this object match the cued one?" and not "was this object presented in the display?" validity doesn't seem like something that can be manipulated.

6. I may have missed it, but it wasn't clear (1) why items were tested twice in the final test phase and what test-phase data (first test, second test, some combination) were used in the analyses and (2) why items were presented on four trials in the

WM phase and whether they were presented in the same condition for each trial or how this information was used when linking the WM conditions/presentations to the LTM data. I know the authors account for the number of presentations, but this may separately influence the results depending on how the repetitions occurred.

7. The cluster based permutation procedure (Page 29) references power values and contralateral/ipsilateral activity which doesn't seem to fit with the rest of the analysis pipeline.

Minor

1. Despite Figure 1, I find the condition labels a bit confusing. At a minimum, I would recommend renaming "Testing" to "Non-prioritization + testing" to both be consistent with the other labels and to avoid some ambiguity -- for instance, on Page 13 Line 23 the word "testing" is used, but I am not sure if that means any testing in WM or literally the "testing" condition as defined in the text. Throughout the text several terms are used (cued, selection, prioritization), I would reduce the terms used/make them more consistent. Rather than "prioritization" and "non-prioritization," "cued" and "non-cued" would be simpler, consistent with prior work, and not assume a mechanism.

2. Are RT analyses restricted to correct trials only?

3. It might be helpful to find a way to incorporate the stats in section 2.4 into Table 2, the section is a bit dense and hard to parse.

4. Figures/Tables. Figure 2C: y-axis has a 0 at the top. Table 2: I would recommend ordering the conditions as they are listed in Figure 1C, rather than have "testing" at the bottom. Figure 5: all of the y-axes are different scales which is confusing. The within learning phase decoding is much greater than the others, but at least the remaining five should be shown on the same scale. The caption has some confusing information (e.g. the y-axis does not depict time) and it's not immediately evident from either the figure labels or the caption what exactly is being decoded.

* TRANSPARENT PEER REVIEW: Communications Psychology uses a transparent peer review system. This means that we publish the editorial decision letters including Reviewers' comments to the authors and the author rebuttal letters online as a supplementary peer review file. However, on author request, confidential information and data can be removed from the published reviewer reports and rebuttal letters prior to publication. If your manuscript has been previously reviewed at another journal, those Reviewers' comments would not form part of the published peer review file.

** Visit Nature Research's author and referees' website at www.nature.com/authors for information about policies, services and author benefits**

Communications Psychology is committed to improving transparency in authorship. As part of our efforts in this direction, we are now requesting that all authors identified as ‘corresponding author’ create and link their Open Researcher and Contributor Identifier (ORCID) with their account on the Manuscript Tracking System prior to acceptance. ORCID helps the scientific community achieve unambiguous attribution of all scholarly contributions. You can create and link your ORCID from the home page of the Manuscript Tracking System by clicking on ‘Modify my Springer Nature account’ and following the instructions in the link below. Please also inform all co-authors that they can add their ORCIDs to their accounts and that they must do so prior to acceptance.
https://www.springernature.com/gp/researchers/orcid/orcid-for-nature-research

For more information please visit http://www.springernature.com/orcid

If you experience problems in linking your ORCID, please contact the Platform Support Helpdesk.

Version 2:

Decision Letter:

** Please ensure you delete the link to your author homepage in this e-mail if you wish to forward it to your coauthors **

Dear Dr Sabo,

Your manuscript titled "Processing in working memory boosts long-term memory representations and their retrieval" has now been seen by our reviewers, whose comments appear below. In light of their advice I am delighted to say that we are happy, in principle, to publish a suitably revised version in Communications Psychology.

We therefore invite you to revise your paper one last time to address the remaining concerns of our reviewers and a list of editorial requests. At the same time we ask that you edit your manuscript to comply with our format requirements and to maximise the accessibility and therefore the impact of your work. The manuscript is overall in good shape, but there are

remaining formatting issues that need to be addressed.

EDITORIAL REQUESTS:

Please review our specific editorial comments and requests regarding your manuscript in the attached "Editorial Requests Table". Please outline your response to each request in the right hand column. Please upload the completed table with your manuscript files as a Related Manuscript file.

If you have any questions or concerns about any of our requests, please do not hesitate to contact me.

SUBMISSION INFORMATION:

In order to accept your paper, we require the files listed here https://www.nature.com/documents/commsj-file-checklist.pdf .

OPEN ACCESS:

Communications Psychology is a fully open access journal. Articles are made freely accessible on publication. For further information about article processing charges, open access funding, and advice and support from Nature Research, please visit https://www.nature.com/commspsychol/open-access

At acceptance, you will be provided with instructions for completing the open access licence agreement on behalf of all authors. This grants us the necessary permissions to publish your paper. Additionally, you will be asked to declare that all required third party permissions have been obtained, and to provide billing information in order to pay the article-processing charge (APC).

* TRANSPARENT PEER REVIEW: Communications Psychology uses a transparent peer review system. On author request, confidential information and data can be removed from the published reviewer reports and rebuttal letters prior to publication. If you are concerned about the release of confidential data, please let us know specifically what information you would like to have removed. Please note that we cannot incorporate redactions for any other reasons.

* CODE AVAILABILITY: All Communications Psychology manuscripts must include a section titled "Code Availability" at the end of the methods section. We require that the custom analysis code supporting your conclusions is made available in a publicly accessible repository at this stage; please choose a repository that generates a digital object identifier (DOI) for the code; the link to the repository and the DOI must be included in the Code Availability statement. Publication as Supplementary Information will not suffice.

* DATA AVAILABILITY:
All Communications Psychology manuscripts must include a section titled "Data Availability" at the end of the Methods section. More information on this policy, is available in the Editorial Requests Table and at http://www.nature.com/authors/policies/data/data-availability-statements-data-citations.pdf.

Please use the following link to submit the above items:
Link Redacted
** This url links to your confidential home page and associated information about manuscripts you may have submitted or be reviewing for us. If you wish to forward this email to co-authors, please delete the link to your homepage first **

We hope to hear from you within two weeks; please let us know if you need more time.

Best regards,

Marike

Marike Schiffer, PhD
Chief Editor
Communications Psychology

REVIEWERS' COMMENTS:

Reviewer #1 (Remarks to the Author):

The authors did a great job in addressing my previous concerns, and I am now happy to recommend the paper for

publication.

Reviewer #2 (Remarks to the Author):

The authors have done an excellent job addressing the previous comments and the manuscript has been substantially strengthened. I have no remaining concerns.

** Visit Nature Research's author and referees' website at www.nature.com/authors for information about policies, services and author benefits**

Open Access This Peer Review File is licensed under a Creative Commons Attribution 4.0 International License, which permits use, sharing, adaptation, distribution and reproduction in any medium or format, as long as you give appropriate credit to the original author(s) and the source, provide a link to the Creative Commons license, and indicate if changes were made.
In cases where reviewers are anonymous, credit should be given to 'Anonymous Referee' and the source.
The images or other third party material in this Peer Review File are included in the article’s Creative Commons license, unless indicated otherwise in a credit line to the material. If material is not included in the article’s Creative Commons license and your intended use is not permitted by statutory regulation or exceeds the permitted use, you will need to obtain permission directly from the copyright holder.
To view a copy of this license, visit https://creativecommons.org/licenses/by/4.0/

<u>EDITOR REPORT:</u>

Thank you for your patience during the peer-review process. Your manuscript titled "Processing in working memory boosts long-term memory representations and their retrieval" has now been seen by 2 reviewers, and I include their comments at the end of this message. They find your work of interest but raised some important points. We are interested in the possibility of publishing your study in Communications Psychology, but would like to consider your responses to these concerns and assess a revised manuscript before we make a final decision on publication.

We therefore invite you to revise and resubmit your manuscript, along with a point-by-point response to the reviewers. Please highlight all changes in the manuscript text file.

Editorially, we consider it critical that both reviewers' requests for additional analyses are met. We also ask that the presentation of the analysis is overall improved, and that any analysis choices (incl preprocessing decisions) are well justified.

We are committed to providing a fair and constructive peer-review process. Please don't hesitate to contact us if you wish to discuss the revision in more detail.
I am attaching an Editorial Requests Table that details critical reporting requirements for the revised manuscript. Please attend to each item and ensure your manuscript is fully compliant. If your revised manuscript is not aligned with these requests on major issues, such as those concerning statistics, it may be returned to you for further revisions without re-review.

Please submit the following items:

- **Revised manuscript**
- **Point-by-point response to the referees' comments**
- **Cover letter (as a separate document)**
- **Nature Research Reporting Summary**
- **Editorial Policy Checklist**
- **Completed Editorial Request Table (attached).**

We would like to thank the Editor, Dr. Marike Schiffer, and the two anonymous reviewers for their thoughtful and constructive feedback. We have carefully addressed all comments and concerns raised by the reviewers, conducted all requested follow-up analyses, and revised the manuscript accordingly. A detailed point-by-point response is provided in this

accompanying reply letter. Below, we summarize the key changes and clarifications made in the revised manuscript:

Revised Interpretation

- Given the reviewers' comments regarding our interpretation, we revised the entire Discussion section to adopt a more balanced view. We now present both the single-trace and multiple-trace accounts as plausible interpretations of our findings, without favoring one too strongly.

Additional Analyses

- In response to Reviewer 1's suggestion, we extended the electrode cluster used in the two main ERP analyses to ensure that no effects were missed; the results remain robust.
- To address concerns about eye movements potentially confounding the decoding results, we performed additional decoding analyses after removing eye movement-related components using independent component analysis and the ICLabel plug-in. These results also replicate the original findings.
- Following Reviewer 2's suggestion, we conducted a regression analysis to test whether decoding performance during the working memory maintenance period predicts subsequent long-term memory performance.

Clarification of Preprocessing and Analysis Choices

- We now clearly explain the rationale for using separate preprocessing pipelines for ERP and decoding analyses, based on established literature (i.e. Carlson et al., 2020).
- To aid transparency and facilitate a quicker understanding of the preprocessing pipeline, we added a figure outlining the full preprocessing workflow and associated EEGLAB functions.
- We clarified the rationale for different downsampling choices (200 Hz vs. 250 Hz) depending on the analysis stage.
- We now describe the trial rejection procedure in more detail, emphasizing that it is based on statistical criteria rather than amplitude thresholds.
- Missing details regarding the EEG setup have been added.
- The cluster-based permutation approach used in the decoding analyses is now described in greater depth.
- We included results from the post-study questionnaire in the Supplementary Materials.

All changes are highlighted in the manuscript and a point-by point reply can be found below. We are grateful for the opportunity to revise and improve our work, and we hope the revised version meets the standards for publication in the journal Communications Psychology.

REVIEWER REPORTS:

Reviewer #1 (Remarks to the Author):

This study addresses an important and timely question - whether working memory can actively modify existing long-term memories. The authors employ a well-structured design in which adult participants learn object-location associations, engage in a working memory task involving attentional prioritization and testing, and later retrieve these associations from long-term memory. The behavioral findings are solid and convincingly demonstrate that both prioritization and testing improve long-term recall, with repeated probes further enhancing performance. The use of EEG combined with decoding analysis to examine shared memory traces across phases is a compelling approach. However, I have some reservations about the EEG-related findings. While the integration of ERP and classification methods is promising, aspects of the methodology are not clearly described, which limits interpretability.

Thank you!

Below, I detail specific issues that require clarification or revision:

## Majors ##

1. Electrode Selection:

- It is unclear why EEG analyses are centered exclusively on three electrodes (P1, P3, and P5). While the LPC is typically observed over left-parietal sites, restricting analyses to only these electrodes may limit sensitivity to broader, potentially relevant activity patterns. A rationale for this choice, or an expanded analysis, would strengthen the conclusions.

Thank you for the suggestion. The electrode choice was motivated by previous findings that suggested a left-parietal modulation during long-term memory retrieval. Following the reviewer's suggestion, we conducted a follow-up analysis, in which we extended the electrode cluster and included the following electrodes: POz, Pz, CPz, PO3, PO4, PO7, PO8, PO9, PO10, P1, P2, P3, P4, P5, P6, P7, P8, P9, P10, CP1, CP2, CP3, CP4. Results of this extended analysis replicate the main results we reported in the manuscript both for Experiment 1 and 2. Specifically, the following significant clusters have been found:

- Experiment 1:
    - Prioritization+testing vs. non-prioritization: 492-600 ms
    - Prioritization+testing vs. absent in working memory: 244-604 ms
- Experiment 2:
    - Prioritization+testing vs. absent in working memory: 220-624 ms
    - Non-prioritization+testing vs. absent in working memory: 308-616ms

- Prioritization+testing vs. non-prioritization: 460-620ms

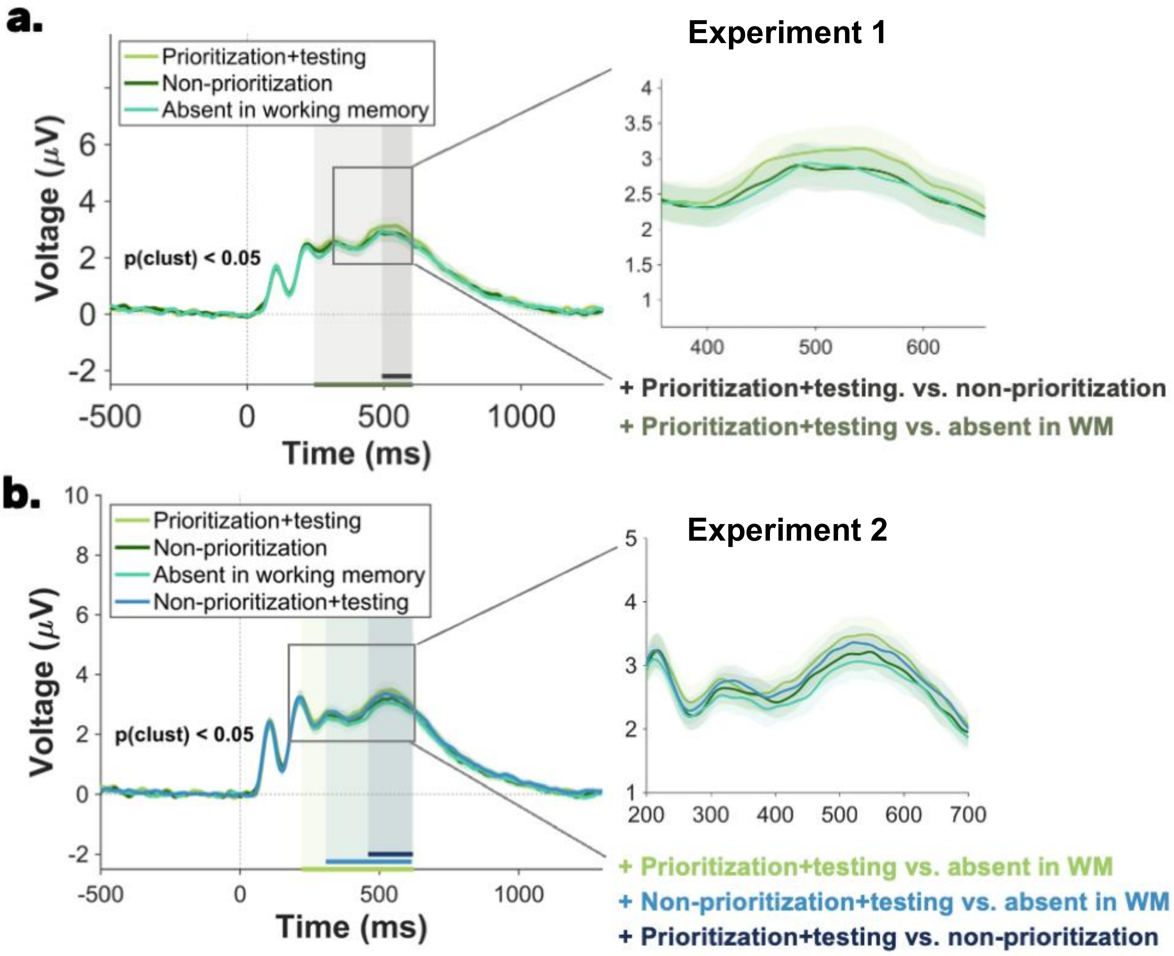

Given that the results are the same, we would prefer to keep the more literature driven approach in the manuscript. Nevertheless, we added the analysis on the extended electrode cluster to the Supplementary materials (see section 5, Figure S3).

- Additionally, there is an inconsistency between the methods and results sections: the methods specify analyses on P1, P3, and P5 (p. 28), yet the results and figures also include Pz. Please clarify which electrodes were analyzed and ensure consistency throughout the manuscript.

Thank you for pointing out the inconsistency regarding the way we report the used electrodes. All our analyses extracted EEG activity from the electrode cluster: Pz, P1, P3, P5 – we made corrections in the manuscript and added Pz, where it was missing.

2. Decoding Feature Source:

The manuscript does not specify where MVPA features were extracted from. Were features limited to the same three electrodes used for ERP analyses? If so, this would significantly constrain the spatial richness MVPA can leverage. Clarification is needed on which channels were included in the classification analysis and how features were defined.

Thank you for pointing out this important omission. We confirm that data from all 64 electrodes were included in the decoding analysis, and the voltage values from these electrodes served as features in the classification procedure. We agree that restricting the analysis to only three electrodes would substantially limit the spatial information available to the classifier, and this was not the case in our analyses.

We have now clarified this point in the Methods section of the revised manuscript to ensure transparency and reproducibility. "Data from all 64 channels served as input features for the classifier." (page 37)

3. Eye Movement Artifacts:
Given the spatial arrangement of visual stimuli, eye movements are likely. However, MVPA analyses appear to include all trials without rejecting those possibly contaminated by ocular artifacts. This raises concerns about the validity of the neural signal. Artifact rejection procedures should be consistently applied across all EEG analyses, including MVPA.
Thank you for raising this important point. We did not apply the same preprocessing pipeline to the MVPA and ERP analyses because prior research has suggested that multivariate methods are relatively robust to noise, including ocular artifacts, and therefore benefit from minimal preprocessing (Carlson et al., 2020). Our preprocessing approach followed the pipeline described by Grootswagers and colleagues (2024), with minor adjustments to suit our specific dataset.

However, we fully agree that potential contamination by eye movements requires careful examination. To address this concern, we reprocessed the EEG data used for the MVPA by incorporating an independent component analysis (ICA) step. Specifically, we applied a 1 Hz high-pass filter as preparation for ICA, then used the ICLabel plug-in to identify and remove components with more than 60% eye-related activity. All other preprocessing steps remained identical.

As shown in the updated Supplementary Materials (section 4), the decoding results remain robust following ocular artifact correction. The key significant time windows are as follows:

- Within phase decoding, encoding phase: 0-996 ms
- Within phase decoding, working memory phase: 44-2224 ms
- Within phase decoding, retrieval phase: 464-1124 ms
- Cross-task decoding, encoding-working memory: 144-496 ms / 444-796 ms
- Cross-task decoding, encoding-retrieval: 172-352 ms / 672-852 ms
- Cross-task decoding, working memory-retrieval: 304-428 ms / 504-728 ms

These results confirm that the decoding effects are not driven by eye movements and strengthen confidence in the validity of the neural signal used in the MVPA.

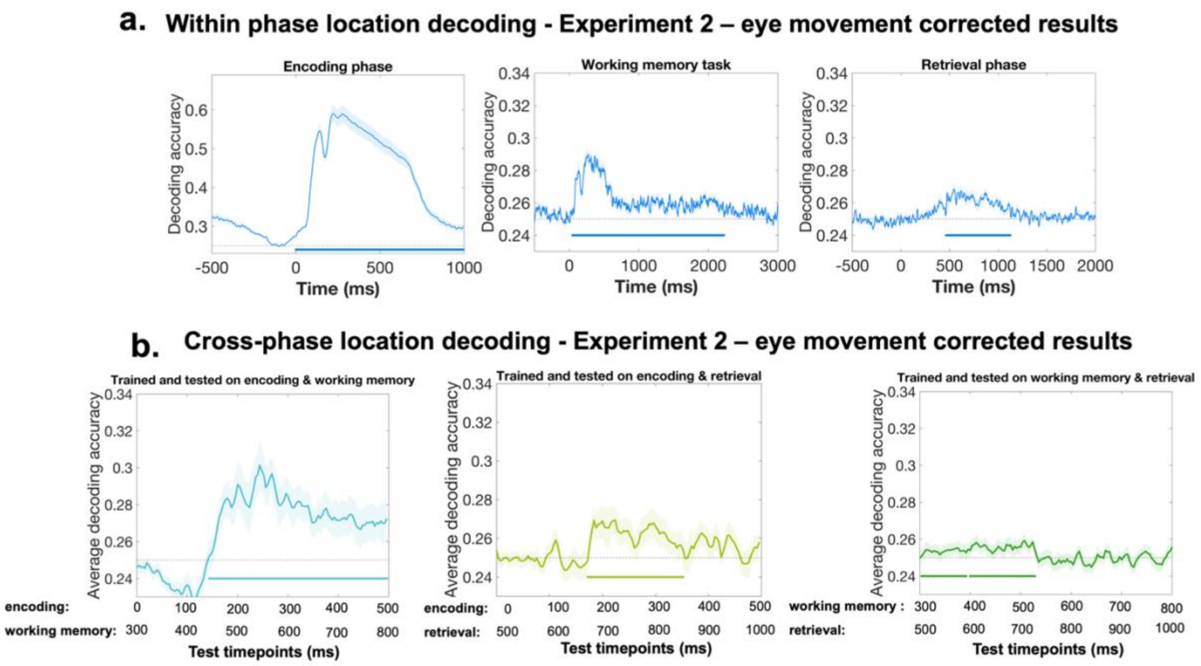

4. Downsampling Discrepancy:

There is an inconsistency regarding the downsampling rate: some sections report downsampling to 200 Hz, while others state 250 Hz. This should be corrected and consistently reported throughout the manuscript.

Thank you for pointing out this inconsistency. We indeed used two different downsampling rates at distinct preprocessing stages. Specifically, the data were downsampled to 200 Hz prior to independent component analysis (ICA) to reduce computation time, while the data used for the final analyses were downsampled to 250 Hz after applying the ICA weights.

To avoid confusion, we have clarified this distinction in the revised manuscript and now explicitly explain the rationale for using two downsampling rates. Additionally, we have added a figure (see Figure 6) illustrating the full preprocessing pipeline, indicating which steps were applied at each stage (e.g., ICA preparation vs. post-ICA processing).

"Preparing for ICA, data were downsampled to 200 Hz to accelerate computation. Importantly, at the end of the preprocessing pipeline, data were downsampled to 250 Hz to retain a higher temporal resolution (see Figure 6)." (page 33)

5. Inconsistent Preprocessing Pipelines:

- The preprocessing steps vary across different analyses. For instance, what is considered an artifact in one analysis seems to be accepted as valid in another. A clearly defined and consistent preprocessing pipeline across analyses is essential.

Thank you for highlighting this important issue. We agree that transparency and clarity in reporting preprocessing procedures are essential. The differences in preprocessing pipelines across analyses were intentional and based on methodological considerations specific to each type of analysis.

In particular, we applied a more extensive artifact rejection procedure for ERP analyses, as these are sensitive to noise. In contrast, minimal preprocessing was applied for the MVPA (decoding) analysis, in line with recommendations from previous studies (Carlson et al., 2020), which suggest that multivariate methods are relatively robust to noise and that excessive preprocessing can distort the multivariate patterns critical for decoding. Our approach is consistent with the pipeline outlined by Grootswagers and colleagues (2024), which we adapted for our data.

The rationale behind the use of a different preprocessing pipeline is explained on page 33 and illustrated in a new figure (Figure 6) outlining the preprocessing flow for each analysis type: "Since Experiment 2 also included a decoding procedure, the preprocessing pipeline has been adapted. Due to the inherent robustness of multivariate methods against noise (Carlson et al., 2020), only minimal preprocessing steps were applied in this case." (page 34)

- While the use of cluster-based permutation tests is well described for ERP analyses, the procedure for statistical evaluation in the decoding analysis is unclear. Did the authors permute category labels during training, testing, or both? Cluster-based permutation testing in classification contexts is not straightforward, and further detail is necessary for interpretation.

Thank you for pointing this out. We used a highly comparable cluster-based permutation approach for both ERP and decoding analyses.

For ERP analyses, we permuted the condition labels across trials. For the decoding analysis, instead of permuting the labels, we generated a null distribution by randomly swapping the actual decoding accuracy values with chance-level values (0.50) within each time point and trial. This approach simulates a null hypothesis where decoding is at chance and was repeated 10,000 times to estimate the distribution of decoding accuracy under the null. Significant clusters were defined as neighboring time points where observed decoding accuracy exceeded the 95th percentile of the null distribution.

We have now clarified the rationale and procedure in the manuscript (page 37). "The only difference in this case was that, instead of permuting condition labels, we generated the null distribution by randomly exchanging observed decoding accuracy values with the chance-level value (0.50) within each trial and time point, which resulted in two vectors. This manipulation was repeated across 10,000 iterations. For each iteration, paired-sample

t-tests were conducted at each time point. Importantly, since we hypothesized that the decoding accuracy is higher than chance level, we applied one-sided t-tests. Clusters of contiguous time points with significant p-values ($p < 0.05$) were then identified, and the size of the largest cluster was recorded. Repeating this process across all permutations yielded a null distribution of maximum cluster sizes, representing the range of cluster sizes expected by chance when decoding performance does not exceed baseline. The 95th percentile of this distribution was used to define the cluster-level threshold for statistical significance. Clusters in the original data that exceeded this threshold were considered statistically significant." (page 37)

6. EEG preprocessing appears to be described in multiple places with varying details, which makes it difficult to follow. Below are specific points that need clarification:
- An artifact detection threshold of 500 µV is unusually high. Why not use the more standard 100 µV?
- The manuscript mentions that a maximum of 5% trial rejection is allowed (line 3, p. 27). What happens when this threshold is exceeded?
Thank you for raising these questions. The EEGLAB function pop_autorej() performs automatic artifact rejection using a probability-based approach. Specifically, it identifies improbable epochs by evaluating their statistical deviation from the overall data distribution. The 'threshold' parameter we set to 500 in our pipeline serves as an initial extreme cutoff for high-amplitude trials—it is not an absolute microvolt-based rejection value. Rather than causing automatic removal of all trials exceeding 500 µV, this value sets an upper boundary to identify which trials should be considered for further probabilistic evaluation. Thus, it is not directly comparable to fixed amplitude thresholds (e.g., 100 µV) often used in simpler artifact rejection approaches.

Using a high threshold such as 500 ensures that the statistical framework has the opportunity to evaluate trials more flexibly, without prematurely excluding data that may not be artifactual. In contrast, applying a lower threshold like 100 µV would risk eliminating trials too early, potentially discarding valuable neural data before the statistical analysis is applied.

The additional parameters 'startprob' defines the number of standard deviations beyond which a trial is initially flagged as a potential outlier, and 'maxrej' controls the maximum number of epochs that can be rejected in a single iteration. In our analysis, both were set to 5, the default values recommended by EEGLAB, which ensures that the rejection process remains adaptive and data-driven across multiple iterations. If the 5% threshold is increased, we risk excluding an excessive amount of trials, this is why in this case, we stick to the default parameter, 5%.

To enhance transparency, we have added the following clarification to the manuscript: "Trials exhibiting extreme fluctuations were then rejected via EEGLAB's automated trial rejection procedure (for an overview, see Table 6) (pop_autorej; 'threshold': 500 µV; 'Startprob': standard deviation value for flagging trials for rejection: 5 standard deviations; 'maxrej': maximum % of rejected trials: 5%). This function performs artifact rejection using a statistical approach that flags improbable epochs based on their deviation from the overall data. The 'threshold' parameter defines a high-amplitude cutoff for flagging candidate trials for rejection. 'Startprob' sets the standard deviation threshold for outliers, and 'maxrej' limits rejections per iteration. This allows for adaptive, data-driven artifact detection beyond fixed amplitude criteria." (page 33)

- The EEG recording setup is not described: What system was used? How many channels were recorded? What was the sampling rate? What were the online filters and referencing procedures?

Thank you for pointing out the missing section. The Methods section has been now updated with the missing information:

"The EEG data were recorded using a passive 64 channel Ag/AgCl system (Easycap GmbH, Herrsching, Germany). The electrodes were distributed according to the extended 10/20 system (CIT). During the EEG recording, a 250 Hz low-pass filter was applied, and the signal was amplified using a NeuroOne Tesla AC-amplifier (Bittium Biosignals Ltd, Kuopio, Finland). The obtained data has a sampling rate of 1000 Hz. FCz served as the reference electrode, while AFz as the ground electrode. During the recording, the impedances were kept below 20 kΩ." (page 31)

- On page 26, Tables 1 and 2 are referenced in the EEG methods section, but these tables contain only behavioral statistics.

Thank you for pointing out this error. All table numbers have been corrected.

- The statement that "anterior channels were excluded from rejection" (p. 26) is unclear. Please specify which channels this refers to.

Thank you for pointing out the missing information. The following channels were excluded from the trial rejection performed before the independent component analysis: Fp1, Fp2, AF3, AF4, AF7, AF8. This information has now been added to the Methods section: "Notably, channels capturing anterior eye movements (i.e., Fp1, Fp2, AF3, AF4, AF7, AF8) were excluded from rejection to optimize eye-movement-related component detection in the subsequent independent component analysis (ICA)." (page 33)

- The baseline period is defined as -200 to 0 ms, but the inter-trial interval during retrieval ranges from 100 to 1500 ms. This raises the possibility that some baselines may include motor-related activity from the previous trial, which should be addressed.

Thank you for pointing this out. This was indeed a typo in the manuscript. The correct inter-trial interval is a randomly jittered duration between 1000 and 1500 ms, not 100–1500 ms as previously stated. Therefore, the baseline period (–200 to 0 ms) should not be affected by motor activity from a previous trial. We have corrected this error in the text and updated Figure 1 accordingly.

7. Interpretation of Cross-Phase Decoding:
While the above-chance cross-phase decoding is intriguing, the conclusion that it reflects memory strengthening or the generation of new traces is not fully supported. An important issue that could strengthen the interpretation would be to assess the extent to which the reactivation findings are trial-specific. However, even if reactivation were shown to be trial-specific, one might still expect that previous and novel memories would not necessarily build on entirely orthogonal representational formats. Although this is an important debate, I am not convinced that the current results provide a strong enough case to resolve this crucial issue in the field.
Thank you for raising this important point.

As our decoding analysis is based on trial-averaged data to improve signal-to-noise ratio, it is unfortunately not well-suited for assessing trial-specific reactivation patterns. We acknowledge that demonstrating trial specificity would provide stronger evidence for memory trace reactivation, but this level of analysis exceeds the limitations of our current dataset and approach.

Regarding the broader theoretical implications, we fully agree that our findings do not provide conclusive evidence to resolve the ongoing debate about whether memory reactivation reflects memory strengthening or the formation of new memory traces. In response to this and related comments from both Reviewer 1 and Reviewer 2, we have revised the manuscript to present a more cautious interpretation. Rather than asserting that our data resolve this theoretical issue, we now frame our results as being consistent with both accounts. See changes on pages 24-25.

Minors
- The discussion section references results from a questionnaire, which appears to have been used to justify the exclusion of one participant. However, this questionnaire is not described anywhere in the methods section. Since data from this instrument were used to inform both the interpretation of results and participant exclusion, it is essential that the authors provide a full description of the questionnaire, including its content, how and when it was administered, and the analysis procedures applied. Additionally, the criteria used to exclude participants

based on this questionnaire should be clearly stated to ensure transparency and reproducibility.

Thank you for raising this important point. We have significantly revised the Discussion section and removed the paragraph referencing the post-study questionnaire. However, we created a new section in the Supplementary materials, in which we report both the questions, as well as participants' responses: "After completing the main task of Experiment 2, participants filled out a post-study questionnaire. They were asked whether they were aware that the same associations were used in the working memory phase as during the initial encoding phase (original German question: *War Ihnen bewusst, dass die zweite Phase auf den Assoziationen der ersten Phase aufbaute?).* Subsequently, they were then asked whether they found this helpful (original German question: *In der zweiten Phase auf einige der Objekt-Positions-Assoziationen aus der ersten Phase zu stoßen, war:*).

For the first question, participants could respond with "yes" or "no." For the second, they could choose from the following four options: (i) useful (*nützlich*), (ii) confusing (*verwirrend*), (iii) neither useful nor confusing (*weder nützlich noch verwirrend*), (iv) I was not aware that the second phase was related to the first (*mir war nicht klar, dass die zweite Phase etwas mit der ersten Phase zu tun hat*). In response to the first question, 32 participants (72.09%) answered "yes," 10 (23.25%) answered "no," and 2 participants (4.65%) did not respond. For the second question, 26 participants (59.09%) found the repetition useful, 3 (6.81%) found it confusing, 11 (25%) indicated it was neither useful nor confusing, and 3 (6.81%) reported being unaware of the connection between the two phases. Overall, these results suggest that most participants recognized the connection between the encoding phase and the working memory task, and that more than half found the repetition useful—indicating an awareness that the objects reappeared on the same locations." (Supplementary materials, section 6, pages 5-6).

Regarding the exclusion of the participant, this decision was not based on the questionnaire, but rather on the study protocol. Specifically, the research assistants, who helped us with the data collection, noted during the experiment that the participant misunderstood the task instructions, and therefore their data were excluded from all analyses. We have now clarified this in the Methods section to improve transparency and reproducibility: "One consistently pressed the right button during the final retrieval phase, due to failure to understand the task instructions, as documented by the research team during data collection in accordance with the study protocol." (see page 27).

Reviewer #2 (Remarks to the Author):

Sabo and colleagues conducted two combined long-term + working memory experiments and recorded EEG in order to assess whether attentional prioritization and testing in working memory (WM) can boost long-term memory (LTM) performance and the associated neural mechanisms. In both experiments,

participants studied object-location pairs, performed a WM task on a subset of the objects, and then performed a location test on the objects. The authors manipulated whether an object was: both cued and tested during the WM phase, not cued but tested during the WM phase, not cued and not tested (but presented) during the WM phase, or not presented during the WM phase. They find that cue+testing out performs the other conditions, including testing without cueing. They find elevated neural signatures of recollection for test probes that were cued and tested. Finally, they find evidence that location information from the study phase is recapitulated during both the WM and test phase. The findings suggest that automatic retrieval of stored information of items entered into WM facilitates later long term recollection.

I find the results of the paper interesting and compelling. The role of WM in LTM formation/retrieval is an active area of interest and I think that this work will make a valuable contribution. I do have some questions/concerns related to some of the conditions and interpretations, but I think that they are all addressable.
Thank you for the positive evaluation of our manuscript.

Major

1. Figure 1 is fantastic for laying out the conditions, but I think that there is an important condition that exists, but was not tested (perhaps due to small trial counts). Specifically, what is the impact on later memory (and the other outcome variables) for items that were prioritized and not tested? For instance, in the case where the selective cue is the horizontal line and the peacock is tested, what happens to memory for the doll? This could provide very interesting information since at the moment it's clear that the combination of prioritization and testing is better than testing alone, but the impact of prioritization alone is unclear.
Thank you for this thoughtful suggestion.

We would first like to clarify a key aspect of our design: whenever an object is cued (i.e., prioritized), participants are required to use the cued object for the match-no-match decision during the working memory test. Therefore, if the reviewer's comment is understood as asking about the effect of prioritization in the complete absence of subsequent testing, we are unable to address this directly—since prioritized items are used to generate a response during the working memory test.

However, a similar question has been addressed in prior work. For instance, Jeanneret and colleagues (2023) employed a 2×2 factorial design to independently manipulate prioritization (prioritized vs. non-prioritized) and testing (tested vs. untested) in working memory and its effect on subsequent long-term memory test. Their findings revealed no

significant interaction between these factors. Although their study differs in some methodological respects—such as the use of reward-based and retroactive cues, and the absence of intentional encoding instructions—the core logic is closely aligned with ours.

Second, we also recognize a different possible interpretation of the reviewer's comment: namely, the interest in what happens to long-term memory performance for the prioritized items when they do not serve as a central probe item during test (e.g., what happens to memory for the doll when the peacock is used as a central probe). Unfortunately, this is another aspect that is not addressable with our current paradigm.

This limitation stems from the structure of our probe presentation. For each trial and object repetition, the identity of the memory probe is randomized: in 50% of cases, it is the cued object (e.g., the doll); in 25%, it is the non-cued item (e.g., the peacock); and in the remaining 25%, it is a novel lure (e.g., a bed). Importantly, each time an object reappears in working memory, the probe can differ. For example, if the doll is cued in one trial and the central probe is the non-cued item (the peacock), the next time the doll is the cued item, the probe might instead be a novel lure. This variability means that we cannot reliably identify a subset of trials in which a prioritized/cued item was never presented as the central probe.

In summary, while our design does not permit a direct test of any effect in question, previous evidence suggests that the lack of testing in working memory might not impact subsequent long-term memory performance.

2. I'm not convinced by the single vs. multi-trace account of how the location information is being represented and I don't think that this interpretation is critical to the paper. The cross-phase decoding indicates that at least some similarly coded location information is shared across phases, but does not rule out the possibility that each phase may additionally contain unique location information.
We appreciate your insightful comment regarding the interpretation of our cross-phase decoding results. We agree that these findings do not provide conclusive evidence for distinguishing between single- versus multi-trace accounts of memory representation.

In response to this and related feedback from both Reviewer 1 and Reviewer 2, we have revised the Discussion section of the manuscript to adopt a more cautious and balanced interpretation. Specifically, we now interpret the cross-phase decoding results as evidence for the neural reinstatement of the location information across phases, and we argue that this is compatible with both the single and the multi-trace account (see changes on pages 24-25).

Furthermore, I don't think that the emphasis on non-consolidated information is warranted, especially given that there is no comparison with presumed

consolidated information. Also, a minor point, but it is not certain that an item presented during the study phase is encoded, thus the current study is not necessarily testing "encoded but not yet consolidated representations."
Thank you for raising this important point. We agree that our original terminology may have been misleading. Our intention was to highlight the distinctive nature of our design, which differs from prior studies where learning occurred incidentally during the working memory task. In contrast, our design involves an initial encoding phase aimed at actively and deliberately learning the material, followed by a separate working memory phase.

However, since we agree that referring to the representations as "encoded but not yet consolidated" may not be fully justified, we have revised the terminology throughout the manuscript. We now refer to these as "newly formed long-term memories" or "existing long-term memories". We hope this revision clarifies our approach and addresses the reviewer's concern (see pages 4, 6, 26).

3. Cross-phase location decoding should fail when applied to the WM maintenance period in the neutral condition in E2, thus it would be beneficial to separate out cued from neutral trials in the analysis/figures. If WM enhances LTM through automatic reinstatement of location information during the WM maintenance phase, then the degree of location information during the WM maintenance phase should predict test performance. The authors should run a regression analysis to test this prediction which would further support their interpretations.
Thank you for the comment. The cross-phase decoding results the reviewer is referring to were conducted on the working memory maintenance period before the presentation of the retro-cue—that is, before any selective cueing occurs. Since this period precedes the retro-cue, processing in the neutral and selective cue conditions should be comparable. Additionally, separating cued and neutral trials in this analysis would be difficult, given the reduction in trial numbers this would entail.

Regarding the second part of the reviewer's comment: Thank you for the valuable analysis suggestion. Following the reviewer's suggestion, we ran a regression analysis relating decoding accuracy averaged during the WM maintenance period (300–800 ms) to participants' accuracy and response times. The results were as follows:
$F(1, 41) = 0.05$, $p = 0.80$ and accounted for approximately 0.15% of the variance in accuracy, $R^2 = 0.0015$.
$F(1, 41) = 1.52$, $p = 0.22$ and accounted for approximately 3.5% of the variance in accuracy, $R^2 = 0.035$.

These findings suggest that decoding accuracy during working memory maintenance does not reliably predict subsequent long-term memory performance. Given the null results and limited explanatory power, we opted not to include these analyses in the manuscript.

4. In a few locations (page 11/ line 16, page 13/line 4) the authors state that their results suggest "a benefit of attention selection over testing," but this could be read as "attention selection (alone) is better than testing (alone)" which is not accurate given that the comparison is between the combination of attention selection and testing vs. testing alone.

Thank you for this comment. We agree that phrasing in scientific writing should be as clear as possible, particularly when describing contrasts between conditions. In our view, the contrast between the *prioritization+testing* condition and the testing-alone condition isolates the added effect of attentional prioritization. We intended the phrase "a benefit of attentional prioritization over testing" to reflect this contrast—namely, the benefit of adding prioritization to testing—rather than to imply that prioritization alone is superior to testing alone.

To avoid any ambiguity, we have revised the relevant phrasing in the manuscript and added clarifications to ensure the intended interpretation is clear to the reader. Specifically, we now explicitly state that the observed effect reflects the added benefit of prioritization when combined with testing and does not imply a superiority of prioritization alone. The relevant revised sections now read as follows: "Comparing this condition to the *prioritization+testing* condition allows us to isolate the added effect of attentional prioritization above and beyond testing in working memory." (pages 10-11)

"However, accuracy was higher in the *prioritization+testing* condition compared to the *non-prioritization+testing* condition, which suggests a benefit of attentional *prioritization* over testing. It is important to note, however, that due to the structure of the experimental design, we are specifically isolating the added benefit of attentional prioritization in conjunction with testing. Therefore, the observed advantage of prioritization over testing should not be interpreted as evidence that prioritization alone is superior to testing alone." (page 13)

5. In the methods (Page 23 / Line 10) cues are described as being 100% valid but it is unclear how the cues could possibly be invalid. Given that the WM test prompt is "does this object match the cued one?" and not "was this object presented in the display?" validity doesn't seem like something that can be manipulated.

Thank you for pointing out this aspect. The reviewer is correct in saying that validity cannot be manipulated in our design, therefore this has now been removed from the manuscript to avoid confusion.

6. I may have missed it, but it wasn't clear (1) why items were tested twice in the final test phase and what test-phase data (first test, second test, some combination) were used in the analyses

Thank you for pointing out this important aspect. Items were tested twice during the final long-term memory retrieval phase to increase the number of available trials per condition, thereby enhancing the signal-to-noise ratio for our EEG analyses and ensuring sufficient statistical power.

To illustrate that both repetitions can be treated equivalently, we compared participants' accuracy across the two tests. Accuracy was highly consistent, with no significant differences:

- Experiment 1: $t(42) = -0.64$, $p = .52$, 95% CI [-0.01, 0.006], $BF_{10} = 0.20$
- Experiment 2: $t(42) = -0.42$, $p = .67$, 95% CI [-0.01, 0.008], $BF_{10} = 0.17$

We included these statistical tests to the Supplementary materials. Additionally, in the revised manuscript, we now clarify both the rationale for including repeated testing and the approach to data inclusion by stating: "Importantly, items were tested twice during the final memory phase to increase trial numbers and improve signal-to-noise ratio. As accuracy did not differ between repetitions (see Supplementary materials), data from both tests were included consistently in all analyses." (page 31 and Supplementary materials section 7, page 6).

(2) why items were presented on four trials in the WM phase and whether they were presented in the same condition for each trial or how this information was used when linking the WM conditions/presentations to the LTM data. I know the authors account for the number of presentations, but this may separately influence the results depending on how the repetitions occurred.

Thank you for raising this important point. Each object was consistently assigned to a specific spatial location and to one working memory condition, and this assignment remained the same across all four repetitions. For example, if participants learned during the encoding phase that a doll was located on the left side, then across all four repetitions in the working memory task, the doll appeared in the same location and under the same condition (e.g., selectively cued). This ensured that the same processes were engaged each time the object was presented in the working memory task.

The reason for presenting each object four times in the working memory phase was to ensure a sufficient number of trials per condition for the electroencephalography analyses. To make this clearer, we have updated Figure 1 and explicitly noted that both the spatial location and the working memory condition were held constant across repetitions.

7. The cluster based permutation procedure (Page 29) references power values and contralateral/ipsilateral activity which doesn't seem to fit with the rest of the analysis pipeline.

Thank you for pointing out this mistake. The reviewer is correct, we did not calculate any literalization index. The mistake has been corrected.

Minor

1. Despite Figure 1, I find the condition labels a bit confusing. At a minimum, I would recommend renaming "Testing" to "Non-prioritization + testing" to both be consistent with the other labels and to avoid some ambiguity -- for instance, on Page 13 Line 23 the word "testing" is used, but I am not sure if that means any testing in WM or literally the "testing" condition as defined in the text. Throughout the text several terms are used (cued, selection, prioritization), I would reduce the terms used/make them more consistent. Rather than "prioritization" and "non-prioritization," "cued" and "non-cued" would be simpler, consistent with prior work, and not assume a mechanism.

Thank you for highlighting this issue. We appreciate the suggestion to clarify and simplify our terminology. To improve consistency and reduce potential ambiguity, we have renamed the "testing" condition to *non-prioritization + testing*, in line with the structure of the other condition labels.

We also reviewed the terminology used throughout the manuscript. We now consistently refer to attentional prioritization as the underlying process of interest, and we have removed references to attentional selection. The terms cued and non-cued are still used, but only when describing the experimental procedure to help readers map onto the task structure more intuitively.

While we acknowledge the reviewer's concern that using the term prioritization could imply a particular mechanism, we decided to retain it when referring to the broader process because it aligns with terminology commonly used in the literature on retro-cue paradigms. Numerous studies over the past two decades have interpreted retro-cue effects in terms of attentional prioritization (for a review, see Souza & Oberauer, 2016), and we believe that using this terminology will help readers better understand the logic behind our design and interpretation.

To clearly differentiate between process-related terms and specific condition labels, we now format all condition labels in italics. We hope this improves the clarity and consistency of our terminology throughout the text.

2. Are RT analyses restricted to correct trials only?

Thank you for raising this issue. No, RT analyses were conducted on all trials. To avoid any misunderstanding, we added the following sentence before reporting RT results: "Notably, all trials were included in the analysis regardless of accuracy." (see pages 9, 18).

3. It might be helpful to find a way to incorporate the stats in section 2.4 into Table 2, the section is a bit dense and hard to parse.

Thank you for the suggestion. We created two new tables, Table 3 and 4 (pages 12 and 14-15) with the results of section 2.4.

4. Figures/Tables. Figure 2C: y-axis has a 0 at the top. Table 2: I would recommend ordering the conditions as they are listed in Figure 1C, rather than have "testing" at the bottom. Figure 5: all of the y-axes are different scales which is confusing. The within learning phase decoding is much greater than the others, but at least the remaining five should be shown on the same scale. The caption has some confusing information (e.g. the y-axis does not depict time) and it's not immediately evident from either the figure labels or the caption what exactly is being decoded.

Thank you for carefully reviewing the figures and pointing out these inconsistencies. We have made the following changes in response to your suggestions:

- We updated Figure 2C.
- Instead of the table, we updated Figure 1C so that the conditions are listed in the order in which they are reported in the Results section.
- The scales of Figure 5 have been updated in line with the reviewer's recommendation.
- The titles in Figure 5 have been updated to highlight that location information is decoded.
- The y-axis depicts decoding accuracy, as in our procedure training and testing is done on a 500 ms long time window. In all three cross-task decoding procedures, data from both phases served as training and testing sets in a reciprocal fashion. For example, in the first panel of Figure 5b, decoding involved training on encoding-phase data (0–500 ms) and testing on working memory data (300–800 ms), and vice versa; the plotted accuracy reflects the average across both directions. Accuracy is shown as a function of testing time. We updated the legend of Figure 5 to clarify how the training and testing have been conducted.

  "Figure 5. Decoding results. (a) Within-phase decoding accuracy for the encoding phase (left), working memory task (middle), and retrieval phase (right). Decoding accuracy is plotted over time relative to stimulus onset, with shaded areas indicating the standard error of the mean. Solid horizontal lines mark significant clusters where accuracy exceeded chance (0.25, given four possible locations), as determined by a cluster-based permutation test: encoding phase (60–996 ms), working memory phase (68–1460 ms), and retrieval phase (368–1420 ms). (b) Cross-phase decoding accuracy for three phase combinations: encoding–working memory (left), encoding–retrieval (middle), and working memory–retrieval (right). In each case, data from both phases served as training and testing sets in a reciprocal fashion. For example, in the left panel, decoding involved training on encoding-phase data (0–500 ms) and testing on working memory data (300–800 ms), and vice versa; the plotted accuracy reflects the average across both

directions. Accuracy is shown as a function of testing time. Each decoding analysis used a 500 ms time window specific to the corresponding phase: encoding (0–500 ms), working memory (300–800 ms), and retrieval (500–1000 ms). Shaded regions represent the standard error of the mean; horizontal lines indicate significant clusters where decoding performance was above chance. Our analysis revealed the following significant clusters: (i) encoding-working memory: 120-496 ms / 420-796; encoding-retrieval: 156-468 ms / 656-968; working memory-retrieval: 300-512 ms / 500-712 ms.“ (page 21)

Bibliography

Carlson, T. A., Grootswagers, T., & Robinson, A. K. (2020). An Introduction to Time-Resolved Decoding Analysis for M/EEG. In D. Poeppel, G. R. Mangun, & M. S. Gazzaniga (Eds.), *The Cognitive Neurosciences* (p. 0). The MIT Press. https://doi.org/10.7551/mitpress/11442.003.0075

Grootswagers, T., Robinson, A. K., Shatek, S. M., & Carlson, T. A. (2024). Mapping the dynamics of visual feature coding: Insights into perception and integration. *PLOS Computational Biology*, *20*(1), e1011760. https://doi.org/10.1371/journal.pcbi.1011760

Jeanneret, S., Bartsch, L. M., & Vergauwe, E. (2023). To be or not to be relevant: Comparing short- and long-term consequences across working memory prioritization procedures. *Attention, Perception, & Psychophysics*, *85*(5), 1486–1498. https://doi.org/10.3758/s13414-023-02706-4

Souza, A. S., & Oberauer, K. (2016). In search of the focus of attention in working memory: 13 years of the retro-cue effect. *Attention, Perception, and Psychophysics*, *78*(7), 1839–1860. https://doi.org/10.3758/s13414-016-1108-5
